# The Stochastic Complexity of Spin Models: Are Pairwise Models Really Simple?

**DOI:** 10.3390/e20100739

**Published:** 2018-09-27

**Authors:** Alberto Beretta, Claudia Battistin, Clélia de Mulatier, Iacopo Mastromatteo, Matteo Marsili

**Affiliations:** 1 The Abdus Salam International Centre for Theoretical Physics (ICTP), Strada Costiera 11, I-34014 Trieste, Italy; 2 Kavli Institute for Systems Neuroscience and Centre for Neural Computation, Norges Teknisk-Naturvitenskapelige Universitet (NTNU), Olav Kyrres Gate 9, 7030 Trondheim, Norway; 3 Department of Physics and Astronomy, University of Pennsylvania, 209 South 33rd Street, Philadelphia, PA 19104-6396, USA; 4 Capital Fund Management, 23 rue de l’Université, 75007 Paris, France; 5 Istituto Nazionale di Fisica Nucleare (INFN) Sezione di Trieste, 34100 Trieste, Italy

**Keywords:** statistical inference, model complexity, minimum description length, spin models

## Abstract

Models can be simple for different reasons: because they yield a simple and computationally efficient interpretation of a generic dataset (e.g., in terms of pairwise dependencies)—as in statistical learning—or because they capture the laws of a specific phenomenon—as e.g., in physics—leading to non-trivial falsifiable predictions. In information theory, the simplicity of a model is quantified by the stochastic complexity, which measures the number of bits needed to encode its parameters. In order to understand how simple models look like, we study the stochastic complexity of spin models with interactions of arbitrary order. We show that bijections within the space of possible interactions preserve the stochastic complexity, which allows to partition the space of all models into equivalence classes. We thus found that the simplicity of a model is not determined by the order of the interactions, but rather by their mutual arrangements. Models where statistical dependencies are localized on non-overlapping groups of few variables are simple, affording predictions on independencies that are easy to falsify. On the contrary, fully connected pairwise models, which are often used in statistical learning, appear to be highly complex, because of their extended set of interactions, and they are hard to falsify.

## 1. Introduction

Science, as the endeavour of reducing complex phenomena to simple principles and models, has been instrumental to solve practical problems. Yet, problems such as image or speech recognition and language translation have shown that Big Data can solve problems without necessarily understanding them [1,2,3]. A statistical model trained on a sufficiently large number of instances can learn how to mimic the performance of the human brain on these tasks [4,5]. These models are simple in the sense that they are easy to evaluate, train, and/or to infer. They offer simple interpretations in terms of low order (typically pairwise) dependencies, which in turn afford an explicit graph theoretical representation [6]. Their aim is not to uncover fundamental laws but to “generalize well”, i.e., to describe well yet unseen data. For this reason, machine learning relies on “universal” models that are apt to describe any possible data on which they can be trained [7], using suitable “regularization” schemes in order to tame parameter fluctuations (overfitting) and achieve small generalization error [8]. Scientific models, instead, are the simplest possible descriptions of experimental results. A physical model is a representation of a real system and its structure reflects the laws and symmetries of Nature. It predicts well not because it generalizes well, but rather because it captures essential features of the specific phenomena that it describes. It should depend on few parameters and is designed to provide predictions that are easy to be falsified [9]. For example, Newton’s laws of motion are consistent with momentum conservation, a fact that can be checked in scattering experiments.

The intuitive notion of a “simple model” hints at a succinct description, one that requires few bits [10]. The *stochastic complexity* [11], derived within Minimum Description Length (MDL) [12,13], provides a quantitative measure for “counting” the complexity of models in bits. The question this paper addresses is: what are the features of simple models according to MDL, and are they simple in the sense surmised in statistical learning or in physics? In particular, are models with up to pairwise interactions, which are frequently used in statistical learning, simple?

We address this issue in the context of spin models, describing the statistical dependence among *n* binary variables. There has been a surge of recent interest in the inference of spin models [14] from high dimensional data, most of which was limited to pairwise models. This is partly because pairwise models allow for an intuitive graph representation of statistical dependencies. Most importantly, since the number of *k*-variable interactions grows as $n^{k}$, the number of samples is hardly sufficient to go beyond k=2. For this reason, efforts to go beyond pairwise interactions have mostly focused on low order interactions (e.g., k=3, see [15] and references therein). Reference [16] recently suggested that even for data generated by models with higher order interactions, pairwise models may provide a sufficiently accurate description of the data. Within the class of pairwise models, L1 regularization [17] has proven to be a remarkably efficient heuristic of model selection (but see also [18]).

Here we focus on the exponential family of spin models with interactions of arbitrary order. This class of models assumes a sharp separation between relevant observables and irrelevant ones, the expected value of which is predicted by the model. In this setting, the stochastic complexity [11] computed within MDL coincides with the penalty that, in Bayesian model selection, accounts for model’s complexity, under non-informative (Jeffrey’s) priors [19].

### 1.1. The Exponential Family of Spin Models (With Interactions of Arbitrary Order)

Consider *n* spin variables, $s=(s_{1},\ldots ,s_{n})$, taking values $s_{i}=\pm 1$ and the set of all product spin operators, $\phi^{\mu }\left(s\right)=\prod_{i\in \mu }s_{i}\;$, where μ is any subset of the indices {1,…,n}. Each operator $\phi^{\mu }\left(s\right)$ models the interaction that involves all the spins in the subset μ.

**Definition** **1.** *The probability distribution of*s*under* spin model M *is defined as:*

(1) $$\begin{array}{cc} & P\left(s\;|\;g,M\right)=\frac{1}{Z_{M}\left(g\right)}e^{\;\sum_{\mu \in M}g^{\mu }\phi^{\mu }\left(s\right)}\;, \end{array}$$

(2) $$\begin{array}{cc} \;\mathrm{with}\;\;\; & Z_{M}\left(g\right)=\sum_{s}e^{\sum_{\mu \in M}g^{\mu }\phi^{\mu }\left(s\right)}\; \end{array}$$

*being the* partition function, *which ensures normalisation. The model* M *is identified by the set* $\left\{\phi^{\mu }\left(s\right),\mu \in \;M\right\}$ *of product spin operators* $\phi^{\mu }\left(s\right)$ *that it contains.*

Note that, under this definition, we consider interactions of arbitrary order (see Section SM-0 of the Supplementary Material). For instance, for pairwise interaction models, the operators $\phi^{\mu }\left(s\right)$ are single spins $s_{i}$ or product of two spins $s_{i}s_{j}$, for i,j∈{1,...,n}. The $g^{\mu }$ are the conjugate parameters [20] that modulate the strength of the interaction associated with $\phi^{\mu }$.

We remark that the model defined in Equation (1) belongs to the *exponential family of spin models*. In other words, it can be derived as the maximum entropy distributions that are consistent with the requirement that the model reproduces the empirical averages of the operators $\phi^{\mu }\left(s\right)$ for all μ∈M on a given dataset [21,22]. In other words, empirical averages of $\phi^{\mu }\left(s\right)$ are sufficient statistics, i.e., their values are enough to compute the maximum likelihood parameters $\hat{g}$. Therefore the choice of the operators $\phi^{\mu }$ in M inherently entails a sharp separation between relevant variables (the sufficient statistics) and irrelevant ones, which may have important consequences in the inference process. For example, if statistical inference assumes pairwise interactions, it might be blind to relevant patterns in the data resulting from higher order interactions. Without prior knowledge, all models M should be compared. According to MDL and Bayesian model selection (see Section SM-0 of the Supplementary Material), models should be compared on the basis of their maximum (log)likelihood penalized by their complexity. In other words, simple models should be preferred a priori.

### 1.2. Stochastic Complexity

The complexity of a model can be defined unambiguously within MDL as the number of bits needed to specify a priori the parameters $\hat{g}$ that best describe a dataset $\hat{s}=\left(s^{\left(1\right)},\cdots ,s^{\left(N\right)}\right)$ consisting of *N* samples independently drawn from the distribution P(s|g,M) for some unknown g (see Section SM-0 of the Supplementary Material). Asymptotically for N→∞, for systems of discrete variables, the *MDL complexity* is given by [23,24]:

(3) $$\log\sum_{\hat{s}}P\left(\hat{s}\;|\;\hat{g},M\right)\;≃\;\frac{\left|M\right|}{2}\log\left(\frac{N}{2\pi }\right)+c_{M}.$$

The first term in the right hand, which is the basis of the Bayesian Information Criterion (BIC) [25,26], captures the increase of the complexity with the number |M| of model’s parameters and with the number *N* of data points. This accounts for the fact that the uncertainty in each parameter $\hat{g}$ decreases with *N* as $N^{-1/2}$, so its description requires $∼\frac{1}{2}\log N$ bits. The second term $c_{M}$ quantifies the statistical dependencies between the parameters, and encodes the intrinsic notion of simplicity we are interested in. The sum of these two terms, in the right hand side of (3), is generally referred as stochastic complexity [11,26].  However, to distinguish these two terms, we will refer to $c_{M}$ as the *stochastic complexity* and the other as BIC term.

**Definition** **2.** *For models of the exponential family, the* stochastic complexity $c_{M}$ *in Equation (3) is given by [23,24]*

(4) $$c_{M}=\log\int dg\;\sqrt{\det J(g)}\;,$$

*where* J(g) *is the Fisher Information matrix with entries*

(5) $$J_{\mu \nu }\left(g\right)=\frac{\partial^{2}}{\partial g^{\mu }\partial g^{\nu }}\log Z_{M}\left(g\right).$$

For the exponential family of models, the MDL criterium (3) coincides with the Bayesian model selection approach, assuming Jeffreys’ prior over the parameters g [26,27,28] (see Section SM-0 of the Supplementary Material). Notice, however, that we take $c_{M}$ as an information theoretic measure of model complexity, and abstain from entering into the debate on whether Jeffreys’ prior is an adequate choice in Bayesian inference (see e.g., [29]).

Within a fully Bayesian approach, the model that maximises its posterior given the data $\hat{s}$, $P(M|\hat{s})$, is the one to be selected. Therefore, if two models have the same number of parameters (same BIC term), the simplest one, i.e., the one with the lowest stochastic complexity $c_{M}$, has to be chosen a priori. However, the number of possible interactions $\phi^{\mu }$ among *n* spins is $2^{n}-1$, and therefore the number of spin models is $2^{2^{n}-1}$. The super-exponential growth of the number of models with the number of spins *n* makes selecting the best model unfeasible even for moderate *n*. Our aim is then to understand how the stochastic complexity depends on the structure of the model M and eventually provide guidelines for the search of simpler models in such a huge space.

## 2. Results

### 2.1. Gauge Transformations

Let’s start by showing that low order interactions do not have a privileged status and are not necessarily related to low complexity $c_{M}$, with the following argument: Alice is interested in finding which model M best describes a dataset $\hat{s}$; Bob is interested in the same problem, but his dataset $\hat{\sigma }=\left(\sigma^{\left(1\right)},\cdots ,\sigma^{\left(N\right)}\right)$ is related to Alice’s dataset by a *gauge transformation*.

**Figure 1:**
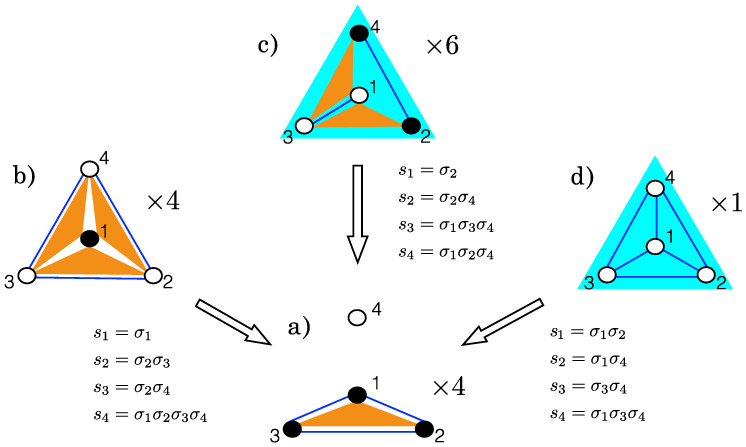
Example of gauge transformations between models with n=4 spins. Models are represented by diagrams (color online): spins are full dots • in presence of a local field, empty dots ∘ otherwise; blue lines are pairwise interactions ($\phi^{\mu }=s_{i}s_{j}$); orange triangles denote 3-spin interactions ($\phi^{\mu }=s_{i}s_{j}s_{k}$); and the 4-spin interaction ($s_{1}s_{2}s_{3}s_{4}$) is a filled blue triangle. Note that model a) has all its interactions grouped on 3 spins; the gauge transformations leading to this model are shown along the arrows. All the models belong to the same complexity class, with |M|=7, λ=4 and a number of independent operators $n_{M}=3$ (e.g., $s_{1},s_{2}$ and $s_{3}$ in model a)—see tables in Section SM-6 of the Supplementary Material. The class contains in total 15 models, which are grouped, with respect to the permutation of the spins, behind the 4 representatives shown here with their multiplicity (×m). Models a), b), c) and d) are respectively identified by the following sets of operators: (**a**) $M=\{s_{1},s_{2},s_{3},s_{1}s_{2},s_{1}s_{3},s_{2}s_{3},s_{1}s_{2}s_{3}\}$; (**b**) $M=\{s_{1},s_{2}s_{3},s_{3}s_{4},s_{4}s_{2},s_{1}s_{2}s_{3},s_{1}s_{3}s_{4},s_{1}s_{2}s_{4}\}$; (**c**) $M=\{s_{2},s_{4},s_{2}s_{4},s_{1}s_{3},s_{1}s_{2}s_{3},s_{1}s_{3}s_{4},s_{1}s_{2}s_{3}s_{4}\}$; and (**d**) $M=\{s_{1}s_{2},s_{1}s_{3},s_{1}s_{4},s_{2}s_{3},s_{2}s_{4},s_{3}s_{4},s_{1}s_{2}s_{3}s_{4}\}$.

**Definition** **3.** *We define a* gauge transformation *as a bijective transformation between n spin variables* $s=\left(s_{1},\cdots ,s_{n}\right)\in {\left\{\pm 1\right\}}^{n}$ *and n spin variables* $\sigma =\left(\sigma_{1},\cdots ,\sigma_{n}\right)\in {\left\{\pm 1\right\}}^{n}$ *that corresponds to a bijection from the set of all operators to itself,* $\phi^{\mu }\left(s\right)\to \phi^{\mu^{\prime }}\left(\sigma \right)$ *(see Section SM-1 of the Supplementary Material). Gauge transformations preserve the structure of the set of all operators. For examples of gauge transformations see Figure 1.*

This gauge transformation induces a bijective transformation between Alice’s models and those of Bob, as shown in Figure 1, that preserves the number of interactions |M|. Whatever conclusion Bob draws on the relative likelihood of models can be translated into Alice’s world, where it has to coincide with Alice’s result. It follows that two models, M and $M^{\prime }$, related by a gauge transformation, must also have the same complexity $c_{M}=c_{M^{\prime }}$. In particular, pairwise interactions can be mapped to interactions of any order (see Figure 1), and, consequently, low order interactions are not necessarily simpler than higher order ones.

**Proposition** **1.** *Two models related by a gauge transformation have the same complexity.*

**Proposition** **2.** *The stochastic complexity* $c_{M}$ *of a model is not defined by the order of its interactions. Models with low order interactions don’t necessarily have a low complexity, and, reciprocally, high order interactions don’t necessarily imply large complexity.*

Observe that models connected by gauge transformations have remarkably different structures. In Figure 1, model a) has all the possible interactions concentrated on 3 spins, having the properties of a simplicial complex [30,31]; however, its gauge-transformed counterparties are not simplicial complexes. Model d) is invariant under any permutations of the four spins, whereas the other models have a lower degree of symmetry under permutations (see the different multiplicities in Figure 1).

Gauge transformations are discussed in more details in Section SM-1 of the Supplementary Material. One can also see them as a change of the basis s→σ in which the operators are expressed. Counting the number of possible bases then gives us the number of gauge transformations (see Section SM-1 of the Supplementary Material).

**Proposition** **3.** *The total number of gauge transformations for a system of n spin variables is:*

(6) $$N_{GT}\left(n\right)=2^{n^{2}}\prod_{k=1}^{n}\left(1-2^{-k}\right).$$

Notice that the number of gauge transformations, (6) is much smaller than the number $2^{n}!$ of possible bijections of the set of $2^{n}$ states into itself. Indeed, a generic bijection between the state spaces of s and σ maps each product operator to one of the binary functions f:σ→{+1,-1}, which does not necessarily correspond to a product operator $\phi^{\mu }\left(\sigma \right)$.

### 2.2. Complexity Classes

Gauge transformations define equivalence relations, which partition the set of all models into equivalence classes. Models belonging to the same class are related to each other by a gauge transformation, and thus, from Proposition 1, have the same complexity $c_{M}$, which leads us to introduce the notion of *complexity classes*.

**Definition** **4.** *A* complexity class *is an equivalence class of models defined by gauge transformations.*

This classification suggests the presence of “quantum numbers” (invariants), in terms of which models can be classified. These invariants emerge explicitly when writing the cluster expansion of the partition function [32,33,34] (see Section SM-2 of the Supplementary Material):

(7) $$Z_{M}\left(g\right)=2^{n}\left(\prod_{\mu \in M}\cosh\left(g^{\mu }\right)\right)\;\sum_{\ell \in L}\;\prod_{\mu \in \ell }\tanh\left(g^{\mu }\right)\;.$$

The sum runs on the set L of all possible *loops ℓ* that can be formed with the operators μ∈M, including the empty loop ℓ=∅.

**Definition** **5.** *A* loop *is any subset* ℓ⊆M *such that* $\prod_{\mu \in \ell }\phi^{\mu }\left(s\right)=1$ *for any value of* s*, i.e., such that each spin* $s_{i}$ *occurs zero or an even number of times in this product.*

The structure of $Z_{M}\left(g\right)$ in (7) depends on few characteristics of the model M: The number |M| of operators (or, equivalently, of parameters) and the structure of its set of loops L (which operator is involved in which loop). The invariance under gauge transformation of the complexity in (4) reveals itself in the fact that the partition function of models related by a gauge transformation have the same functional dependence on their parameters up to relabeling.

**Figure 2:**
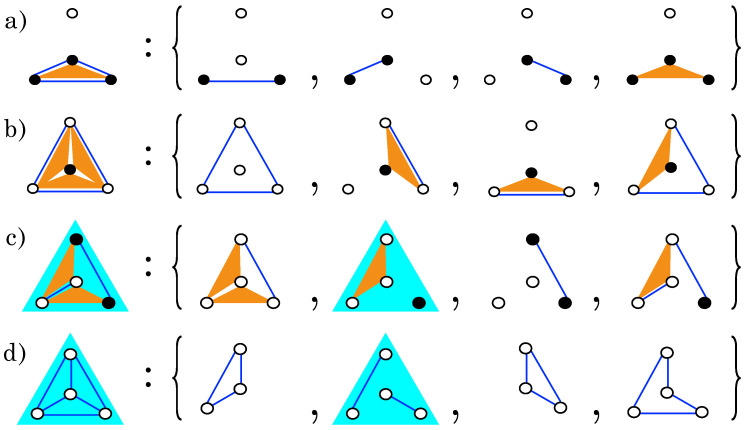
Example of a minimal generating set of loops for each model of Figure 1. As these models belong to the same class, their (respective) sets of loops have the same cardinality, $2^{\lambda }$, where λ=4 is the number of generators (as shown here). For model a), one can easily check that the 4 loops of the set are independent, as each of them contains at least one operator that doesn’t appear in the other 3 loops (see Section SM-3 of the Supplementary Material). Within each column, on the r.h.s., loops are related by the same gauge transformation morphing models into one another on the l.h.s. (i.e., the transformations displayed in Figure 1). This shows that the loops of these 4 generating sets have the same structure, which implies that the loop structure of the 4 models is the same. Any loop of a model can finally be obtained by combining a subset of its generating loops. Note that the choice of the generating set is not unique. (**a**–**d**) refer to the same models as in Figure 1.

Let us focus on the loop structure of models belonging to the same class. The set L of loops of any model M has the structure of a finite Abelian group: if $\ell_{1},\ell_{2}\in L$, then $\ell_{1}⊕\ell_{2}$ is also a loop of M, where ⊕ is the symmetric difference [35] of two sets (see Section SM-3 of the Supplementary Material). As a consequence, for each model M one can identify a *minimal generating set* of λ loops, such that any loop in L can be uniquely expressed as the symmetric difference of loops in the minimal generating set. Note that the choice of the generating set is not unique, though all choices have the same cardinality λ; Figure 2 gives examples of this decomposition for the models of Figure 1. Note also that ℓ⊕ℓ=∅ for each loop ℓ∈L. As a consequence, the cardinality of the loop group is $\left|L\right|=2^{\lambda }$ (including the empty loop ∅). We found that λ is related to the number |M| of operators of the model by $\lambda =\left|M\right|-n_{M}$ (see Section SM-3 of the Supplementary Material), where $n_{M}$ is the number of *independent operators* of a model M, i.e., the maximal number of operators that can be taken in M without forming any loop. This implies that λ attains its minimal value, λ=0, for models with only independent operators ($\left|M\right|=n_{M}$), and its maximal value, $\lambda =2^{n}-1-n$, for the *complete model* $\bar{M}$, that contains all the $|\bar{M}|=2^{n}-1$ possible operators. The number of independent operators, $n_{M}$, is preserved by gauge transformation, and, as the total number of operators, |M|, is also an invariant of the class, so is the cardinality of the minimal generating set λ. For example, all models in Figure 1 have $n_{M}=3$ independent operators and λ=4 (see Figure 2). It can also be shown that gauge transformations imply a duality relation, that associates to each class of models with |M| operators a class of models with the $2^{n}-1-\left|M\right|$ complementary operators (see Section SM-3 of the Supplementary Material).

To summarize, the distinctive features of a complexity class are given in the following Proposition.

**Proposition** **4.** *The number of operators,* |M| *, the number of independent operators,* $n_{M}$ *, and the structure of the set of loops,* L *(through its generators), fully characterize a complexity class.*

## 3. Discussion: How Do Simple Models Look Like?

### 3.1. Fewer Independent Operators, Shorter Loops

Coming to the quantitative estimate of the complexity, $c_{M}$ generally depends on the extent to which ensemble averages of the operators $\phi^{\mu }\left(s\right)$ in the model μ∈M constrain each other. This appears explicitly by rewriting (4) as an integral over the ensemble averages of the operators, $\varphi =\{\langle \phi^{\mu }\rangle ,\mu \in M\}$, exploiting the bijection between the parameters g and their dual parameters φ and re-parameterization invariance [28,36]:

(8) $$c_{M}=\log\int_{F}d\varphi \sqrt{\det J(\varphi )}\;,\;$$

where J(φ) is the Fisher Information Matrix in the φ-coordinates. The new domain, F, of integration is over the values of φ that can be realized in any empirical sample drawn from the model M (known in this context as *marginal polytope* [37,38]) and is related to the mutual constraints between the ensemble averages, $\varphi^{\mu }$, (see Section SM-4 of the Supplementary Material for more details).

**Proposition** **5.** *The complexity of a model without loops, i.e.,*L={∅}*, and*$n_{M}=\left|M\right|$*independent operators is*$c_{M}=\left|M\right|\log\pi$.

Indeed, if the model contains no loop, then $J_{\mu \nu }\left(\varphi \right)={\left[1-{\left(\varphi^{\mu }\right)}^{2}\right]}^{-1}\delta_{\mu \nu }$ is diagonal: The integral in (8) factorizes and Proposition 5 follows. In this case, the variables $\varphi^{\mu }$ are not constrained at all and the domain of integration is $F={\left[-1,1\right]}^{\left|M\right|}$. If instead the model contains loops, the variables $\varphi^{\mu }$ become constrained and the marginal polytope, F, is reduced. For example, for a model with a single loop of length three (e.g., $\phi^{1}=s_{1}$, $\phi^{2}=s_{2}$, and $\phi^{3}=s_{1}s_{2}$), the values of φ in ${\left[-1,1\right]}^{3}$ are not all attainable, indeed $F=\{\varphi \in {\left[-1,1\right]}^{3}:\;|\varphi^{1}+\varphi^{2}|-1\leq \varphi^{3}\leq 1-|\varphi^{1}-\varphi^{2}|\}$ is reduced, which decreases the complexity.

**Proposition** **6.** *The complexity of models with a fixed number of operators and a single (non-empty) loop increases with the length of the loop.*

**Figure 3:**
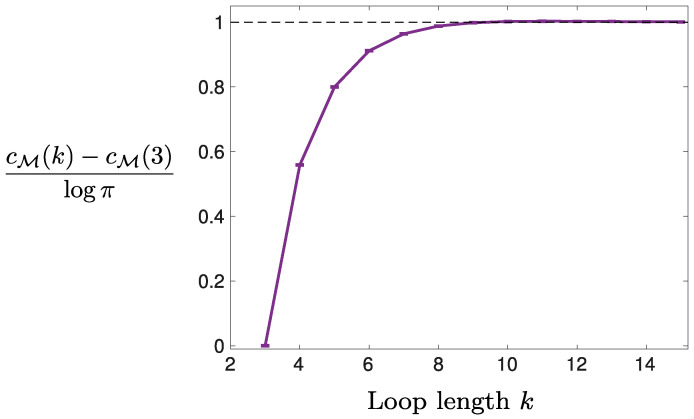
Complexity, $c_{M}\left(k\right)$, of models with a single loop of length *k*, and |M|-k *free* operators, i.e., not involved in any loop. For k=3, $c_{M}\left(3\right)=\left(|M|-1\right)\log\pi$ can be computed analytically from (4). Values of $c_{M}$ are averaged over $10^{3}$ numerical estimates of the integral in (4), using $10^{6}$ Monte Carlo samples each. Error bars correspond to their standard deviation.

The complexity, $c_{M}\left(k\right)$, of models with a fixed number, |M|, of parameters and a single (non-empty) loop of length *k* is shown in Figure 3 (see Section SM-6 of the Supplementary Material): $c_{M}\left(k\right)$ increases with *k* and saturates at |M|logπ, which is the value one would expect if all operators were unconstrained. This is consistent with the expectation that longer loops induce weaker constraints among the operators. Note that the number of independent operators is kept constant here, equal to $n_{M}=\left|M\right|-1$.

**Proposition** **7.** *At fixed number of operators, the complexity of a model increases with the number of independent operators.*

**Figure 4:**
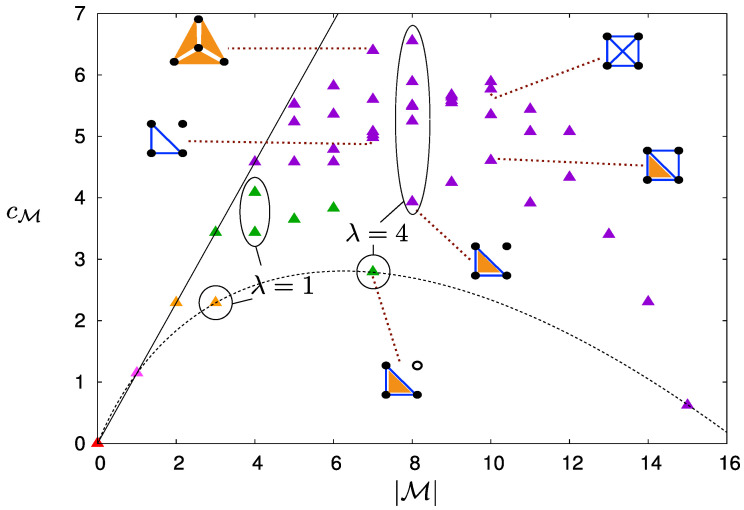
(color online) Complexity of models for n=4 as a function of the number |M| of operators: Each triangle represents a class of complexity, which contains one or more models (see Section SM-6 of the Supplementary Material). For each class, the value of $c_{M}$ was obtained from a representative of the class; some of them are shown here with their corresponding diagram (same notation as in Figure 1). The triangle colors indicate the values of $n_{M}$: Violet for $n_{M}=4$, green for 3, yellow for 2, pink for 1, and red for 0 (model with no operator). Models on the black line have only independent operators ($\left|M\right|=n_{M}$) and complexity, $c_{M}=\left|M\right|\;\log\pi$; models on the dashed curve are complete models, the complexities of which are given in (9). Complexity classes with the same values of |M| and $n_{M}$ have the same value of $\lambda =\left|M\right|-n_{M}$, i.e., the same number of loops, |L|, but a different loop structure.

The single loop calculation allows computing the complexity of models with non-overlapping loops ($\ell \cap \ell^{\prime }=\emptyset$ for all $\ell ,\ell^{\prime }\in L$), for which $c_{M}=\sum_{\ell \in L}c_{\ell }$ is the sum over the complexity, $c_{\ell }$, associated to each loop. In the general case of models with more complex loop structures, the explicit calculation of $c_{M}$ is non-trivial. Yet, the argument above suggests that, at fixed number of parameters |M|, $c_{M}$ should increase with the number $n_{M}$ of independent operators. Figure 4 summarises the results for all models with n=4 spins and supports this conclusion: For a given value of |M|, classes with lower values of $n_{M}$ (i.e., with less independent operators) are less complex.

**Proposition** **8.** *At fixed number of operators, complete models on a subset of spins and their equivalents are the simplest models. We refer to these models as* sub-complete models. *Classes of sub-complete models are the* classes of minimal complexity.

A surprising result of Figure 4 is that $c_{M}$ is not monotonic with the number, |M|, of operators of the model, increasing first with |M| and then decreasing. Complete models $\bar{M}$ turn out to be the simplest (see the dashed curve in Figure 4). As a consequence, for a given |M|, models that contain a complete model on a subset of spins are generally simpler than models where operators have support on all the spins. For instance, the complexity class displayed in Figure 1 is the class of models with |M|=7 operators that has the lowest complexity (see green triangle on the dashed curve in Figure 4).

Figure 4 also confirms that pairwise models are not simpler than models with higher order interactions. Indeed, for instance for |M|=7, $c_{M}$ increases drastically when changing model a) of Figure 1 into a pairwise model by turning the 3-spin interaction into an external field acting on $s_{4}$. Likewise, the model with all six pairwise interactions for |M|=10 is more complex than the one where one of them is turned into a 3-spin interaction.

### 3.2. Complete and Sub-Complete Models

It is possible to compute explicitly the complexity of a complete model, $\bar{M}$, with *n* spins using the mapping $g^{\mu }=2^{-n}\sum_{s}\phi^{\mu }\left(s\right)\log p\left(s\right)$ between the $2^{n}-1$ parameters $g^{\mu }$ of $\bar{M}$ and the $2^{n}$ probabilities p(s) constrained by their normalization [39]. The complexity in (4) being invariant under reparametrization [36], one can easily re-write this integral in terms of the variables p(s). Finally, using that $\det J\left(p\right)=\prod_{s}1/p\left(s\right)$, we obtain the following proposition (see Section SM-5 of the Supplementary Material).

**Proposition** **9.** *The complexity of a complete model* $\bar{M}$ *with n spins is:*

(9) $$\begin{array}{ccc} c_{\bar{M}} & = & \log\int_{0}^{1}\;dp\;\delta \left(\sum_{s}p\left(s\right)-1\right)\prod_{s}\frac{1}{\sqrt{p(s)}}\;,\\ & = & 2^{n-1}\log\pi -\log\Gamma \left(2^{n-1}\right)\;. \end{array}$$

Note that, for n>4, $c_{\bar{M}}$ becomes negative (for n=6, $c_{\bar{M}}≃-41.5$). This suggests that the class of least complex models with |M| interactions is the one that contains the model where the maximal number of loops are concentrated on the smallest number of spins. This agrees with our previous observations on single loop models and sub-complete models. On the contrary, models where interactions are distributed uniformly across the variables (e.g., models with only single spin operators for n≥|M| or with non-overlapping sets of loops) have higher complexity.

We finally note that complete models are also extremely simple to infer from empirical data. Indeed, the maximum likelihood estimates of the parameter are trivially given by ${\hat{g}}^{\mu }=2^{-n}\sum_{s}\phi^{\mu }\left(s\right)\log\hat{p}\left(s\right)$, where $\hat{p}\left(s\right)$ is the empirical distribution. By contrast, learning the parameters of pairwise interacting models can be a daunting task [14].

### 3.3. Maximally Overlapping Loops

This finally leads us to conjecture that stochastic complexity is related to the localization properties of the set of loops L (i.e., its group structure) rather than to the order of the interactions: Models where the loops, $\ell ,\ell^{\prime }\in L$, have a “large” overlap, $\ell \cap \ell^{\prime }$, are simple, whereas models with an extended homogeneous network of interactions (e.g., fully connected Ising models with up-to pairwise interaction) have many non-overlapping loops, $\ell \cap \ell^{\prime }=\emptyset$, and therefore are rather complex. It is interesting to note that the former (simple models) lend themselves to predictions on the independence of different groups of spins. These predictions suggest “fundamental” properties of the system under study (i.e., invariance properties, spin permutation symmetry breaking) and are easy to falsify (i.e., it is clear how to devise a statistical test for these hypotheses to any given confidence level). On the contrary, complex models (e.g., fully connected pairwise Ising models) are harder to falsify as their parameters can be adjusted to fit reasonably well any sample, irrespectively of the system under study.

### 3.4. Summary

We find that at fixed number, |M|, of operators, simpler models are those with fewer independent operators (i.e., smaller $n_{M}$). For the same value of $n_{M}$, models can still have different complexities. The simpler ones are then those with a loop structure that will impose the most constraints between the operators of the model. More generally, we show that the complexity of a model is not defined by the order of the interactions involved, but is, instead, intimately connected to its internal geometry, i.e., how interactions are arranged in the model. The geometry of this arrangement implies mutual dependencies between interactions that constrain the states accessible to the system. More complex models are those that implement fewer constraints, and can thus account for broader types of data. This result is consistent with the information geometric approach of Reference [26], which studies model complexity in terms of the geometry of the space of probability distributions [40]. The contribution of this paper clarifies the relation between the information geometric point of view and the specific structure of the model, i.e., the actual arrangement of its interactions. We remark that these results apply to non-degenerate spin models. In the broader class of degenerate models [20], arrangement of the operators and degeneracy of the parameters may interact non trivially in terms of complexity. Our preliminary numerical investigation of single loop models (see Section SM-7 of the Supplementary Material) indicates that degeneracy decreases complexity by constraining the parameters and therefore the statistics of the operators. Also, Reference [41] discusses model selection on mixture models of spin variables, and shows that they can be cast in the form of Equation (1), where $g^{\mu }$ are subject to linear constraints. For these models, Bayesian model selection can be performed exactly without resorting to the approach discussed here.

A rough estimate of the number, *N*, of data samples beyond which the complexity term becomes negligible in Bayesian inference can be obtained with the following argument: An upper bound for the complexity of models with *n* spins and *m* parameters is given by mlogπ, i.e., when all operators are independent. As a lower bound, we take Equation (9) with $m=2^{n}-1$. This implies that an upper bound for the variation of the complexity is given by $\Delta c=\frac{m-1}{2}\log\pi +\log\Gamma \left(\frac{m+1}{2}\right)$. When this is much smaller than the BIC term, the stochastic complexity can be neglected. For large *m* this implies N≫m, which may be relevant for the applicability of fully connected pairwise models ($m≃n^{2}/2$) in typical cases, for instance when samples cannot be considered as independent observations from a stationary distribution (see [18]).

## 4. Conclusions

As pointed out by Wigner [42] long ago, the unreasonable effectiveness of mathematical models relies on isolating phenomena that depend on few variables, whose mutual variation is described by simple models and is independent of the rest. Remarkably we find that, for a fixed number of spin variables and parameters, simple models, according to MDL, are precisely of this form: Statistical dependencies are concentrated on the smallest subset of variables and these are independent of all the rest. Such simple models are not optimal for generalizing, i.e., to describe generic statistical dependencies, rather they are easy to falsify. They are designed for spotting independencies that may hint at deeper principles (e.g., symmetries or conservation laws) that may “take us beyond the data” [43], meaning that they can hint at hypotheses (on e.g., symmetries or other regularities) that can be tested in future experiments. On the contrary, fully connected pairwise models, which provide simple interpretation of statistical dependencies in terms of direct interactions, appears to be rather complex. This, we conjecture, is the origin of pairwise sufficiency [16] that makes them so successful to describe a wide variety of data from neural tissues [44] to voting behaviour [45].

Our results, however, show that any model that can be obtained from a pairwise model via a gauge transformation has the same complexity and hence the same generalisation power, but has higher order interactions. Hence, gauge transformations can be used to compare pairwise models with models in the same complexity class, in order to quantitatively assess when a dataset is genuinely described by pairwise interactions. Notice that this comparison can be done directly on the basis of their maximum likelihood alone.

This is only one of the possible applications of gauge transformations, which are one of the main results of this paper. In loose words, these transformations preserve the topology of models, i.e., the manner in which interactions are mutually arranged, but change the “basis” of the operators that embody these interactions. Besides model selection within the same complexity class, as in the example of pairwise models above, we can think of selecting the appropriate complexity class according to the availability of data. One particularly interesting avenue of future research is to perform model selection among models of minimal complexity (i.e., sub-complete models). Model comparison between and within these classes may be relevant given the high degree of clustering and modularity in neural, social, metabolic, and regulatory networks [46]. These models offer the simplest possible explanation of a dataset, not necessarily the most accurate one (e.g., in terms of generalisation error), but the one that can potentially reveal regularities and symmetries in the data. Interestingly, parameter inference for these models is also remarkably simple.

In conclusion, when data are scarce and high dimensional, Bayesian inference should privilege simple models, i.e., those with small stochastic complexity, over more complex ones, such as fully connected pairwise models that are often used [14,44,45]. A full Bayesian model selection approach is hampered by the calculation of the stochastic complexity that is a daunting task. Developing approximate heuristics for accomplishing this task is a challenging future avenue of research.

## Supplementary Materials

The Supplementary Material is available online at http://www.mdpi.com/1099-4300/20/10/739/s1.
