# Supplementary material for: The Stochastic Complexity of Spin Models: Are Pairwise Models Really Simple?
Source: Entropy (Basel). 2018 Sep 27;20(10):739. doi: 10.3390/e20100739 (PMC7512302; doi:10.3390/e20100739)
Supplement: Supplementary file 1 [file entropy-20-00739-s001.pdf]

Supplementary Materials: The Stochastic Complexity of Spin Models: Are Pairwise Models Really Simple?

Alberto Beretta, Claudia Battistin, Clélia de Mulatier, Iacopo Mastromatteo and Matteo Marsili

SM-0. General Framework–Spin models, Stochastic Complexity

SM-0.1. Spin Operators

Let us consider the system of n spin variables, $\mathbf{s} = (s_1, \cdots, s_n)$, that take random values $s_i = \pm 1$. In order to account for interactions of any order, we associate to any interaction involving a subset μ of spins a *spin operator* $\phi^\mu(\mathbf{s})$ defined as the product of all the spins in μ:

$$\phi^\mu(\mathbf{s}) = \prod_{i \in \mu} s_i\,, \tag{S.1}$$

which takes value in $\{+1, -1\}$. By definition, the total number of these operators corresponds to the number of possible interactions in the n-spin system, i.e. to the number of possible subsets of $\{s_1, \ldots, s_n\}$, empty set excluded, which is $2^n - 1$. In the following we simplify the notation of the operator label μ by using an integer, $\mu \in \{1, \ldots, 2^n - 1\}$, whose binary representation directly identifies the spins that belong to the set μ [1]. For instance, the operator $\phi^1(\mathbf{s}) = s_1$ is associated with a field acting on s_1, and $\phi^7(\mathbf{s}) = s_1 s_2 s_3$ with a three body interaction. These *spin operators* are the building blocks of the models. Note that, these operators verify:

$$\sum_{\mathbf{s} \in \mathcal{S}} \phi^\mu(\mathbf{s}) = 0\,, \qquad \mu \in \{1, \ldots, 2^n - 1\}\,, \tag{S.2}$$

where $\mathcal{S} = \{-1, 1\}^n$, and the sum over $\mathbf{s} \in \mathcal{S}$ denotes the sum over all possible configurations of the spins.

SM-0.2. Complete Set of Spin Operators

We define the set $\Omega_n = \{\phi^\mu(\mathbf{s})\}_{\mu \in \{0,\ldots,2^n-1\}}$ of all the spin operators built with n spins, including also the operator $\phi^0(\mathbf{s}) = 1$ (which is not associated with any interaction). By definition, the cardinality of Ω_n is $|\Omega_n| = 2^n$. The set Ω_n is (called) *orthogonal* and *complete* as its operators verify respectively the relations [2]:

$$\langle \phi^\mu(\mathbf{s}), \phi^\nu(\mathbf{s}) \rangle = \frac{1}{2^n} \sum_{\mathbf{s} \in \mathcal{S}} \phi^\mu(\mathbf{s})\, \phi^\nu(\mathbf{s}) = \delta_{\mu,\nu} \qquad \text{and} \qquad \frac{1}{2^n} \sum_{\mu=0}^{2^n-1} \phi^\mu(\mathbf{s})\, \phi^\mu(\mathbf{s}') = \delta_{\mathbf{s},\mathbf{s}'}. \tag{S.3}$$

The first relation defines an inner product $\langle \cdot, \cdot \rangle$ over the space of operators Ω_n. The relation derives from the fact that the product of two operators of Ω_n is also an operator of Ω_n:

$$\phi^\mu(\mathbf{s})\, \phi^\nu(\mathbf{s}) = \phi^{\mu \oplus \nu}(\mathbf{s})\,, \tag{S.4}$$

where, in the binary representation of μ and ν, $\oplus$ is the XOR bitwise operation. Using the property (S.2) and observing that $\phi^\mu(\mathbf{s})\phi^\mu(\mathbf{s}) = \phi^0(\mathbf{s}) = 1$ gives the first relation. The second relation is an immediate consequence of the fact that, for the set of monomials Ω_n, one has

$$\sum_{\mu=0}^{2^n-1} \phi^\mu(\boldsymbol{s})\,\phi^\mu(\boldsymbol{s}') = \sum_{\alpha_1=0,1} \cdots \sum_{\alpha_n=0,1} \prod_{i=1}^{n} (s_i s_i')^{\alpha_i}\,, \tag{S.5}$$

which is always equal to zero, unless all s_i are equal to s_i'. In this latter case the sum yields the 2^n factor that allows to recover (S.3). The orthogonality and completeness properties (S.3) allow us to express any function $F : \mathcal{S} \to \mathbb{R}$ [3] as a linear combination of operators [2] $\phi^\mu(\boldsymbol{s})$, i.e.,

$$F(\boldsymbol{s}) = \sum_{\mu\in\Omega_n} f^\mu \phi^\mu(\boldsymbol{s}), \qquad \text{and} \qquad f^\mu = \langle F(\boldsymbol{s}), \phi^\mu(\boldsymbol{s})\rangle = \frac{1}{2^n}\sum_{\boldsymbol{s}\in\mathcal{S}} \phi^\mu(\boldsymbol{s}) F(\boldsymbol{s}). \tag{S.6}$$

Generating Set of Ω_n and Independent Operators

In the following, we will call *generating set* of Ω_n a set of n spin operators that can fully generate Ω_n, such as the set $\{s_1, \ldots, s_n\}$. We also define the notion of *set of independent operators* [4] as a set $\mathcal{I}$ verifying that the product of all the operators of any subset of $\mathcal{I}$ is always different from $\phi^0(\boldsymbol{s}) = 1$. Formally, any set of n *independent operators* of Ω_n is a *generating set* of Ω_n. By definition, a generating set of Ω_n cannot include the identity operator ϕ^0.

Mathematically, the set Ω_n, associated with the multiplication operation $(\Omega_n, \cdot)$, forms a finite Abelian group with identity element $\phi^0(s) = 1$ generated by a minimal set of n generators of order 2 ($\Omega_n = {\mathbb{Z}_2}^n$).

SM-0.3. Spin Models

A *model* $\mathcal{M}$ is defined in terms of a subset $\mathcal{M} \subseteq \Omega_n \backslash \{\phi^0\}$ of operators [5]. These define a probability distribution of the vector $\boldsymbol{s} = (s_1, \ldots, s_n)$ of spin variables:

$$P(\boldsymbol{s}\,|\,\boldsymbol{g}, \mathcal{M}) = \frac{1}{Z_{\mathcal{M}}(\boldsymbol{g})} e^{\sum_{\mu\in\mathcal{M}} g^\mu \phi^\mu(\boldsymbol{s})} \qquad \text{where} \qquad Z_{\mathcal{M}}(\boldsymbol{g}) = \sum_{\boldsymbol{s}\in\mathcal{S}} e^{\sum_{\mu\in\mathcal{M}} g^\mu \phi^\mu(\boldsymbol{s})}\,, \tag{S.7}$$

where the vector $\boldsymbol{g} = \{g^\mu,\ \mu \in \mathcal{M}\}$ are the conjugate parameters: each parameter g^μ is a real variable that modulates the strength of the interaction associated with the operator $\phi^\mu(\boldsymbol{s})$. We shall refer to the model $\bar{\mathcal{M}} = \Omega_n \backslash \{\phi^0\}$ with all operators as the *complete model*. Models can be *degenerate* (several operators are mapped to the same parameter) or not.

Non-Degenerate Models

Non-degenerate models are those for which each operator $\phi^\mu(\boldsymbol{s})$ is assigned a different parameter g^μ. For instance, model a) in Figure 1 of the main text involves $|\mathcal{M}| = 7$ interactions, mapping the 7 operators $\mathcal{M} = \{s_1, s_2, s_3, s_1 s_2, s_1 s_3, s_2 s_3, s_1 s_2 s_3\}$ onto the 7 parameters $\boldsymbol{g} = \{g^1, g^2, g^4, g^3, g^5, g^6, g^7\}$ (using the binary representation [1] of μ). The number of different non-degenerate models with n spins grows superexponentially in n:

$$\mathcal{N}_n = 2^{|\Omega_n \backslash \{1\}|} = 2^{2^n - 1}\,. \tag{S.8}$$

To give an idea: $\mathcal{N}_2 = 8$, $\mathcal{N}_3 = 128$, $\mathcal{N}_4 = 32768$, $\mathcal{N}_5 \simeq 2 \cdot 10^9$. In the main paper and in most of the supplemental material we shall focus on non-degenerate models.

Degenerate Models

For completeness we also define *degenerate models*, which are discussed in section SM-7. In a *degenerate model*, each parameter can be associated to one or more interactions. For example, the mean

field Ising model is a degenerate model with only 2 parameters, h and J; the connection with the g^μ notation reads:

$$g^\mu = \begin{cases} h & \text{for all } \mu = 2^k \quad \text{with } k \in [0, n-1], \\ J & \text{for all } \mu = 2^k + 2^{k'} \quad \text{with } k > k' \text{ and } (k, k') \in [0, n-1]^2, \\ 0 & \text{otherwise} \quad , \end{cases} \tag{S.9}$$

where we used the binary representation of the set μ [1]. To work with degenerate models, it is convenient to introduce a more general notation, in which a model $\mathcal{M}$ is defined by a set of $|\mathcal{M}|$ operators, $\boldsymbol{\phi} = \{\phi^\mu\}_{\mu\in\mathcal{M}}$, a set of m parameters $\boldsymbol{g} = \{g^i\}_{i\in\{0,\dots,m\}}$, and a rectangular (mapping) matrix $\mathbb{U}$ of size $|\mathcal{M}| \times m$ that maps each operator of $\boldsymbol{\phi}$ to one parameter of $\boldsymbol{g}$:

$$U_{ij} = \begin{cases} 1 & \text{if } \phi^j \text{ is parameterised by } g^i, \\ 0 & \text{otherwise}. \end{cases} \tag{S.10}$$

For non-degenerate models, $\mathbb{U}$ is simply the $|\mathcal{M}| \times |\mathcal{M}|$ identity matrix. By definition, each column of $\mathbb{U}$ contains a single 1, whereas the sum of each line i gives the degeneracy $\alpha_i = \sum_j U_{ij}$ of the parameter g^i. Note that $\sum_{ij} U_{ij} = |\mathcal{M}|$. The extension to such *degenerate* models is natural when operators $\mu \in \mathcal{V}$ are of the same order [6]. The number of possible degenerate models, where interactions of the same order may be assigned the same parameter, grows much faster than $\mathcal{N}_n$ with n:

$$\mathcal{N}_n^{deg} = \prod_{j=1}^{n} B_{\binom{n}{j}+1},$$

where B_m is the number of partitions of a set with m elements, known as Bell number. For instance, $\mathcal{N}_2^{deg} = 10$, $\mathcal{N}_3^{deg} = 450$, $\mathcal{N}_4^{deg} = 2.371.408$ and $\mathcal{N}_5^{deg} \simeq 38 \cdot 10^{15}$.

SM-0.4. The Stochastic Complexity and Bayesian Model Selection

In this section we recall the relation between the *stochastic complexity* defined in the context of Minimum Description Length and the *geometric complexity* obtained from a Bayesian approach. We refer to References [7,8] for a more complete treatment.

Bayesian model selection dictates that, given a dataset $\hat{\boldsymbol{s}} = (\boldsymbol{s}^{(1)}, \dots, \boldsymbol{s}^{(N)})$ of N observed configurations $\boldsymbol{s}^{(i)} \in \mathcal{S}$, each model should be assigned a posterior probability

$$P(\mathcal{M} \,|\, \hat{\boldsymbol{s}}) = \frac{P(\hat{\boldsymbol{s}} \,|\, \mathcal{M})\, P_0(\mathcal{M})}{\sum_{\mathcal{M}'} P(\hat{\boldsymbol{s}} \,|\, \mathcal{M}')\, P_0(\mathcal{M}')}, \tag{S.11}$$

according to Bayes' rule. Here $P_0(\mathcal{M})$ is the prior probability on the model $\mathcal{M}$ and the sum in the denominator runs on all models $\mathcal{M}'$ that are considered. In Equation (S.11), $P(\hat{\boldsymbol{s}}|\mathcal{M})$ is the so-called evidence that is computed by integrating the likelihood $P(\hat{\boldsymbol{s}} \,|\, \boldsymbol{g}, \mathcal{M})$ over the parameters $\boldsymbol{g}$. In the case where $\boldsymbol{s}^{(i)}$ are i.i.d., this reads:

$$P(\hat{\boldsymbol{s}} \,|\, \mathcal{M}) = \int \mathrm{d}\boldsymbol{g} \prod_{i=1}^{N} P(\boldsymbol{s}^{(i)} \,|\, \boldsymbol{g}, \mathcal{M})\; P_0(\boldsymbol{g}|\mathcal{M}) \tag{S.12}$$

where $P_0(\boldsymbol{g}|\mathcal{M})$ is the prior distribution on the parameters $\boldsymbol{g}$ of model $\mathcal{M}$. For spin models, the probability $P(\boldsymbol{s} \,|\, \boldsymbol{g}, \mathcal{M})$ is given by Equation (S.7) and the evidence becomes:

$$P(\hat{\boldsymbol{s}} \,|\, \mathcal{M}) = \int \mathrm{d}\boldsymbol{g}\; \mathrm{e}^{N\, [\hat{\boldsymbol{\varphi}}(\hat{\boldsymbol{s}}) \cdot \boldsymbol{g} \,-\, \log Z_{\mathcal{M}}(\boldsymbol{g})]}\; P_0(\boldsymbol{g} \,|\, \mathcal{M}), \tag{S.13}$$

where $\hat{\boldsymbol{\varphi}}(\hat{\boldsymbol{s}})$ is a vector with $|\mathcal{M}|$ elements, containing the empirical averages of the operators ϕ^μ over the measured data $\hat{\boldsymbol{s}}$:

$$\hat{\varphi}^\mu(\hat{\boldsymbol{s}}) = \frac{1}{N}\sum_{i=1}^{N}\phi^\mu(\boldsymbol{s}^{(i)}), \qquad \text{for } \mu \in \mathcal{M}. \tag{S.14}$$

The log-likelihood, $\log P(\hat{\boldsymbol{s}}\,|\,\boldsymbol{g},\mathcal{M}) = N\,[\hat{\boldsymbol{\varphi}}(\hat{\boldsymbol{s}})\cdot\boldsymbol{g} - \log Z_{\mathcal{M}}(\boldsymbol{g})]$, is a convex function of $\boldsymbol{g}$ and it has a unique maximum for the values of the parameters $\boldsymbol{g} = \hat{\boldsymbol{g}}$ that are the solution of the set of equations:

$$\varphi^\mu(\hat{\boldsymbol{g}}) = \hat{\varphi}^\mu(\hat{\boldsymbol{s}}) \qquad \text{for all } \mu \in \mathcal{M}, \tag{S.15}$$

where

$$\varphi^\mu(\boldsymbol{g}) = \frac{\partial \log Z_{\mathcal{M}}(\boldsymbol{g})}{\partial g^\mu} = \sum_{s\in\mathcal{S}}\phi^\mu(\boldsymbol{s})\;P(\boldsymbol{s}\,|\,\boldsymbol{g},\mathcal{M}), \tag{S.16}$$

denotes the ensemble average of the operator $\phi^\mu(\boldsymbol{s})$ under the model specified by $\boldsymbol{g}$. In other words, at $\boldsymbol{g} = \hat{\boldsymbol{g}}$, the ensemble average of each operator ϕ^μ of $\mathcal{M}$ is equal to its empirical average $\hat{\varphi}^\mu$ over the measured data $\hat{\boldsymbol{s}}$. For large N, the integral is sharply dominated by the maximum and it can be estimated by the Saddle-point method, expanding $\hat{\boldsymbol{\varphi}}(\hat{\boldsymbol{s}})\cdot\boldsymbol{g} - \log Z_{\mathcal{M}}(\boldsymbol{g})$ to second order about $\hat{\boldsymbol{g}}$. This shows that, for large N, Equation (S.13) is well approximated by:

$$\log P(\hat{\boldsymbol{s}}\,|\,\mathcal{M}) \cong \log P(\hat{\boldsymbol{s}}\,|\,\hat{\boldsymbol{g}},\mathcal{M}) - \frac{|\mathcal{M}|}{2}\log\left(\frac{N}{2\pi}\right) - c_{\mathcal{M}}^{\mathrm{BMS}} + O\left(\frac{1}{N}\right), \tag{S.17}$$

where $c_{\mathcal{M}}^{\mathrm{BMS}}$ is a geometric complexity term [8] arising from the Gaussian integration:

$$c_{\mathcal{M}}^{\mathrm{BMS}} = \log\left[\frac{\sqrt{\det \mathbb{J}(\hat{\boldsymbol{g}})}}{P_0(\hat{\boldsymbol{g}}\,|\,\mathcal{M})}\right], \tag{S.18}$$

and $\mathbb{J}(\boldsymbol{g})$ is the Hessian of the log-likelihood, which in this case coincides with the Fisher Information matrix defined in Equation (5) of the main text.

Minimum Description Length instead approaches the problem of model complexity from an apparently different angle. Imagine we run a series of experiments that generate a sample $\hat{\boldsymbol{s}}$ of $N \gg 1$ observations of a system. We model the outcome of the experiment as N i.i.d. drawn from a model $P(\boldsymbol{s}\,|\,\boldsymbol{g},\mathcal{M})$ for unknown parameters $\boldsymbol{g}$ (imagine the situation where we run the experiment precisely because we want to infer the parameters $\boldsymbol{g}$). How much memory storage should be set aside *before* running the experiment? If we knew the parameters $\hat{\boldsymbol{g}}$ the solution is given by (minus) the log-likelihood $-\log P(\hat{\boldsymbol{s}}\,|\,\hat{\boldsymbol{g}},\mathcal{M})$ where $P(\hat{\boldsymbol{s}}|\ldots) = \prod_{i=1}^{N} P(\boldsymbol{s}^{(i)}|\ldots)$. In the absence of this information, the problem can be cast as a minimax problem (we refer to [9] for details), i.e., to find the best possible coding $\bar{P}(\hat{\boldsymbol{s}})$ in the case where Nature choses the worst possible sample $\hat{\boldsymbol{s}}$. The solution is the *normalised maximum likelihood*

$$\bar{P}(\hat{\boldsymbol{s}}) = \frac{P(\hat{\boldsymbol{s}}\,|\,\hat{\boldsymbol{g}}(\hat{\boldsymbol{s}}),\mathcal{M})}{\sum_{\hat{\boldsymbol{s}}'} P(\hat{\boldsymbol{s}}'\,|\,\hat{\boldsymbol{g}}(\hat{\boldsymbol{s}}'),\mathcal{M})}. \tag{S.19}$$

From this, it is clear that the additional memory space that is needed to describe the model and the parameters is given by the log of the denominator of Equation (S.19), which is the l.h.s. in Equation (3) of the main text. In order to derive the r.h.s. of Equation (3) of the main text, consider the expansion:

$$\int dg\, P(\hat{s}\,|\,g,\mathcal{M})\, f(g) \simeq P(\hat{s}\,|\,\hat{g},\mathcal{M})\, f(\hat{g})\, \frac{(2\pi/N)^{|\mathcal{M}|/2}}{\sqrt{\det \mathbb{J}(\hat{g})}}\, [1+O(1/N)] \tag{S.20}$$

that arises from performing the integral by saddle point around the maximum likelihood parameters $\hat{g}(\hat{s})$ which depend on the data $\hat{s}$. In Equation (S.20), the matrix $\mathbb{J}$ is, in general, the Hessian of the likelihood at $\hat{g}$. Yet, for exponential models, the Hessian $\mathbb{J}$ does not depend on the data, and it coincides with the Fisher Information matrix. Taking $f(g) = \sqrt{\det \mathbb{J}(g)}$, summing over all samples $\hat{s}$ in Equation (S.20) and taking the limit $N \to \infty$, one finds

$$\int dg \sqrt{\det \mathbb{J}(g)} = \lim_{N\to\infty} \left(\frac{2\pi}{N}\right)^{|\mathcal{M}|/2} \sum_{\hat{s}} P(\hat{s}\,|\,\hat{g},\mathcal{M}) = e^{c_{\mathcal{M}}}\,, \tag{S.21}$$

which is Equation (3) of the main text, and where $c_{\mathcal{M}}$ is given by Equation (4) of the main text.

As observed in Reference [8], the choice of *Jeffreys' priors* [10]

$$P_0(g\,|\,\mathcal{M}) = \frac{\sqrt{\det \mathbb{J}(g)}}{\int dg' \sqrt{\det \mathbb{J}(g')}} \tag{S.22}$$

in Equation (S.18) makes the geometric complexity $c_{\mathcal{M}}^{\mathrm{BMS}}$ of the Bayesian approach coincide with the stochastic complexity $c_{\mathcal{M}}$ (see Equation (4) of the main text) prescribed by Minimum Description Length [7,11]. This choice for the prior seems natural (in absence of any information on the values of g), as it corresponds to assuming an *a priori* uniform distribution in the space of samples [8]. We will see that this choice of prior has also an interesting property, as it is invariant under re-parametrisation, which will lead to the definition of class of complexity.

SM-1. Gauge Transformations (GT)

SM-1.1. Definition

Any generating set $\sigma = \{\phi^{\nu_1},\ldots,\phi^{\nu_n}\}$ of Ω_n induces a bijection $s \to \sigma(s)$ on the set of configurations $\mathcal{S}$ and on the set Ω_n of operators. Indeed

$$\phi^{\mu}(\sigma) = \prod_{i\in\mu} \phi^{\nu_i}(s) = \phi^{\mu'}(s), \qquad \mu' = \oplus_{i\in\mu}\nu_i \tag{S.23}$$

where $\oplus_{i\in\mu}\nu_i$ is the bitwise XOR of the binary representation of the integers ν_i for all $i \in \mu$. We call such a bijection a *gauge transformation* [12]. In other words, these are transformations that map the set of n generators $\{s_1,\ldots,s_n\}$ of Ω_n to another set of generators of Ω_n, i.e. a set of *n independent operators* of Ω_n (see definitions in SM-0). A GT *preserves the structure* of Ω_n in the sense that any operator in the old basis is mapped into a distinct operator in the new one. A transformation that maps $(s_1,\ldots,s_n)$ to a set of n non-independent operators will not preserve its structure. Indeed it maps Ω_n to a strict subset of Ω_n, with $n' < n$ independent generators. Combining them can generate only $2^{n'}$ operators, which means that some operators of Ω_n will not occur in $\Omega_{n'}$. Note also that the operator $\phi^0(s) = 1$ is invariant under GTs.

Mathematically, these transformations are the *automorphisms* of the group $(\Omega_n,\cdot)$.

SM-1.2. Number of Gauge Transformations for A System with n Spins

The total number of these transformations corresponds to the number of possible sets of generators of Ω_n. There are exactly

$$\binom{|\Omega_n|-1}{n} \times n! = \prod_{i=1}^{n}(2^n - i) = (2^n)^n \prod_{i=1}^{n}\left(1-\frac{i}{2^n}\right) \simeq (2^n)^n \left(1+O(n^2/2^n)\right) \tag{S.24}$$

possible ways to sample a set of n operators among $\Omega_n \backslash \{1\}$ [13] . However, only a few of them correspond to a set of n *independent operators*. Consider that you have chosen i independent operators, $\{\sigma_1, \ldots, \sigma_i\}$, in $\Omega_n \backslash \{1\}$: with these operators you can generate a subset of 2^i operators of Ω_n. The number of operators left in Ω_n that are independent of the family $\{\sigma_1, \ldots, \sigma_i\}$ is thus $|\Omega_n| - 2^i = 2^n - 2^i$, which corresponds to the number of possibilities for choosing another independent operator σ_{i+1}. As a consequence, the number of different ways to sample n independent operators from Ω_n, i.e., the total number of GTs, is

$$\mathcal{N}_{GT}(n) = \prod_{i=0}^{n-1} (2^n - 2^i) = (2^n)^n \prod_{i=1}^{n} \left(1 - \frac{1}{2^i}\right) . \quad \text{(S.25)}$$

In this equation, one can recognise the q-Pochhammer symbol, $\left(\frac{1}{2}, \frac{1}{2}\right)_n = \prod_{i=0}^{n-1} (1 - (\frac{1}{2})^{i+1})$, a (strictly) decreasing function of n, converging rapidly ($n > 5$) to its asymptotic value known as the Euler ϕ-function, $\phi(\frac{1}{2}) \simeq 0.2887880950$. For example, $\mathcal{N}_{GT}(3) = 168$ and $\mathcal{N}_{GT}(4) = 20160$; for $n > 5$, the number of gauge transformations grows as $\mathcal{N}_{GT}(n) \sim 0.289 \times (2^n)^n$. Finally, the probability of getting a GT by drawing at random n operators $\{\sigma_1, \ldots, \sigma_n\}$ of Ω_n converges asymptotically to a non-zero constant:

$$\mathcal{P}_{GT} = \frac{\left(\frac{1}{2}, \frac{1}{2}\right)_n}{\prod_{i=1}^{n} \left(1 - \frac{i}{2^n}\right)} \underset{n \to \infty}{\longrightarrow} \phi\left(\frac{1}{2}\right) \simeq 0.2887880950 . \quad \text{(S.26)}$$

SM-2. Partition Function of A Spin Model $\mathcal{M}$

SM-2.1. Partition Function and Loops of $\mathcal{M}$

In order to compute the complexity $c_{\mathcal{M}}$ of a model $\mathcal{M}$ from Equation (4) of the main text, one has first to compute the Fisher Information matrix $\mathbb{J}(\boldsymbol{g})$, and, by extension, the partition function $Z_{\mathcal{M}}(\boldsymbol{g})$ given in Equation (S.7). As each operator $\phi^\mu(\boldsymbol{s})$ only takes values in $\{-1, 1\}$, the exponential terms in Equation (S.7) can be expanded as [14,15]

$$e^{g^\mu \phi^\mu(\boldsymbol{s})} = \cosh(g^\mu) + \phi^\mu(\boldsymbol{s}) \sinh(g^\mu) = \cosh(g^\mu) \left[1 + \phi^\mu(\boldsymbol{s}) \tanh(g^\mu)\right] , \quad \text{(S.27)}$$

which successively leads to the expressions for the partition function:

$$\begin{aligned} Z_{\mathcal{M}}(\boldsymbol{g}) &= \left(\prod_{\mu \in \mathcal{M}} \cosh(g^\mu)\right) \sum_{\boldsymbol{s} \in \mathcal{S}} \prod_{\mu \in \mathcal{M}} \left[1 + \phi^\mu(\boldsymbol{s}) \tanh(g^\mu)\right] , \\ &= \left(\prod_{\mu \in \mathcal{M}} \cosh(g^\mu)\right) \sum_{\boldsymbol{s} \in \mathcal{S}} \left[\sum_{\mathcal{M}' \subseteq \mathcal{M}} \prod_{\mu \in \mathcal{M}'} \phi^\mu(\boldsymbol{s}) \tanh(g^\mu)\right] , \end{aligned}$$

where the sum over $\mathcal{M}' \subseteq \mathcal{M}$ runs over all possible *sub-models* (i.e., subsets) of $\mathcal{M}$ and the product is then taken over every operator of the sub-model $\mathcal{M}'$. The "empty model" $\mathcal{M}' = \{\varnothing\}$, with no interactions, is also included in the sum, considering that $\prod_{\mu \in \{\varnothing\}} \phi^\mu \tanh(g^\mu) = 1$. In order to compute the sum over all configurations $\mathcal{S}$, one can exploit Equations (S.2) and (S.4), that lead to:

$$\begin{aligned} \sum_{\boldsymbol{s} \in \mathcal{S}} \prod_{\mu \in \mathcal{M}'} \phi^\mu(\boldsymbol{s}) \tanh(g^\mu) &= \prod_{\mu \in \mathcal{M}'} \tanh(g^\mu) \sum_{\boldsymbol{s} \in \mathcal{S}} \phi^{\oplus_{\mu \in \mathcal{M}'}}(\boldsymbol{s}) \\ &= 2^n \prod_{\mu \in \mathcal{M}'} \tanh(g^\mu) \delta_{\oplus_{\mu \in \mathcal{M}'}, 0} , \end{aligned}$$

where $\oplus_{\mu \in \mathcal{M}'}$ denotes the bitwise XOR operation between all the operators $\mu \in \mathcal{M}'$. Here, the key observation is that $\oplus_{\mu \in \mathcal{M}'} = 0$ if and only if each spin occurs an even number of times (or none) among the operators of $\mathcal{M}'$. In this latter case, the operators of $\mathcal{M}'$ form a *loop*, such that $\prod_{\mu \in \mathcal{M}'} \phi^{\mu}(s) = 1$ is equal to the identity operator. Let us name ℓ any sub-model $\mathcal{M}'$ that forms a *loop* and call $\mathcal{L}$ the set of all the loops ℓ of a given model $\mathcal{M}$ (including the empty loop $\{\emptyset\}$), allowing us to obtain the expression in Equation (7) of the main text. The expansion of the partition function in loops is in the same spirit of cluster expansions methods in statistical physics (for a review see [16]).

SM-2.2. Invariance of $Z_{\mathcal{M}}$ under Gauge Transformation

In Equation (7) of the main text, the structure of the partition function depends only on few characteristics of the model $\mathcal{M}$:

i) the total number of operators $|\mathcal{M}|$, as they all appear in the product $\prod_{\mu \in \mathcal{M}} \cosh(g^{\mu})$;
ii) the structure of its set of *loops* $\mathcal{L}$: the number $|\mathcal{L}|$ of loops in the model (through the sum over $\mathcal{L}$); the number $|\ell|$ of operators involved in each loop, named the *length of the loop* (through the product over each operator μ of ℓ); and finally which operators are involved in each loop.

These properties are invariant under GTs, such that the structure of the partition function in Equation (7) of the main text remains invariant as well. Indeed, consider two models, $\mathcal{M}$ and $\mathcal{M}' = \mathcal{T}[\mathcal{M}]$, that are images of one another via a GT $\mathcal{T}$. They verify the following properties:

i) the two models have the same number of operators: $|\mathcal{M}| = |\mathcal{M}'|$. Indeed, we define the image of the set $\mathcal{M}$ by $\mathcal{T}$ as, $\mathcal{M}' = \mathcal{T}[\mathcal{M}] = \{\mathcal{T}[\phi^{\mu}], \mu \in \mathcal{M}\}$, and $\mathcal{T}$ is a bijection on the set of operators Ω_n (such that for all ϕ^{μ}, $\mathcal{T}[\phi^{\mu}] \in \Omega_n$ and if $\phi^{\mu} \neq \phi^{\nu}$ then $\mathcal{T}[\phi^{\mu}] \neq \mathcal{T}[\phi^{\nu}]$).
ii) the two models have the same loop structure. Indeed, if $\ell \in \mathcal{L}$ is a loop of the model $\mathcal{M}$, i.e.,

$$\prod_{\mu \in \ell} \phi^{\mu}(s) = \phi^{\oplus_{\mu \in \ell}} = 1,$$

then $\ell' = \mathcal{T}(\ell)$ has the same length as ℓ (see i)) and is a loop of the model $\mathcal{M}' = \mathcal{T}[\mathcal{M}]$:

$$\prod_{\mu \in \ell'} \phi^{\mu}(s) = \prod_{\mu \in \ell} \mathcal{T}[\phi^{\mu}(s)] = \mathcal{T}[\phi^{0}(s)] = 1,$$

where we used that $\mathcal{T}[\phi^{\mu}(s) \cdot \phi^{\mu'}(s)] = \mathcal{T}[\phi^{\mu}(s)] \cdot \mathcal{T}[\phi^{\mu'}(s)]$ (as $\mathcal{T}$ is an homomorphism of Ω_n), and that the identity element ϕ^0 of Ω_n is invariant under $\mathcal{T}$. Reciprocally, as $\mathcal{T}$ is a bijection, if ℓ' is a loop of $\mathcal{M}'$ then $\ell = \mathcal{T}^{-1}[\ell']$ is a loop of $\mathcal{M}$. Finally, if ℓ_1 and ℓ_2 are two distinct loops of $\mathcal{M}$, then their respective images by $\mathcal{T}$ are two distinct loops of $\mathcal{M}'$.
In other words, if $\mathcal{L}$ is the group of loops of the model $\mathcal{M}$, then the group of loops associated to the model $\mathcal{M}' = \mathcal{T}[\mathcal{M}]$ is $\mathcal{L}' = \mathcal{T}[\mathcal{L}]$.

As a consequence, if two models are related by a GT, $\mathcal{M}' = \mathcal{T}[\mathcal{M}]$, then they have the same value of complexity $c_{\mathcal{M}} = c_{\mathcal{M}'}$. Indeed, the function under the integral in Equation (4) of the main text stays invariant under the change of variables from the model $\mathcal{M}$ to the model $\mathcal{M}'$. Finally, gauge transformations define an equivalence relation between models, for which the structure (previously described, see *i)* and *ii)*) and the complexity $c_{\mathcal{M}}$ are invariant. Gauge transformations thus allow us to partition all models into equivalence classes, that we call *complexity classes*. For instance, Figure 1 of the main text displays several models for $n = 4$ that belong to the same *complexity class* (highlighted in bold font in Table S2) for which $c_{\mathcal{M}} \simeq 2.8$. Note that, conversely, $c_{\mathcal{M}} = c_{\mathcal{M}'}$ does not imply that $\mathcal{M}$ and $\mathcal{M}'$ belong to the same complexity class. For example, the models $\mathcal{M} = \{s_1, s_2\}$ and $\mathcal{M}' = \{s_1, s_2, s_1 s_2\}$ have both $c_{\mathcal{M}} = c_{\mathcal{M}'} = 2 \log \pi$ (see Table S1), but their structures are clearly different.

SM-3. Complexity Classes and Loop Structure of Spin Models

Let us highlight several interesting properties of models belonging to the same class of complexity. First, the *number of independent operators* $n_{\mathcal{M}}$ in a model $\mathcal{M}$ is invariant under GT and is thus a property of each complexity class (see SM-3.1). It can besides be (strictly) smaller than the system size n: any model with $n_{\mathcal{M}} < n$ is equivalent to a model involving only $n_{\mathcal{M}}$ spins. Second, the set of loops $\mathcal{L}$ of a model (including the empty loop $\{\varnothing\}$) has the structure of a finite group, from which we show that the total number of loops of a given model $\mathcal{M}$ is of the form (see SM-3.2):

$$|\mathcal{L}| = 2^{\lambda} \qquad \text{with} \;\; \lambda = |\mathcal{M}| - n_{\mathcal{M}} \,. \tag{S.28}$$

The structure of the group $\mathcal{L}$ is an invariant of the class. Table S1 gives, for instance, a description of the loop structure for each class of complexity of models with $n = 3$. Finally, GTs also imply a duality relation between complexity classes of *complementary models*: each class of models with $|\mathcal{M}|$ operators corresponds to a *complementary class* of models with $(2^n - 1) - |\mathcal{M}|$ operators that contains the same number of models (see SM-3.3 and Table S1).

SM-3.1. Number of Independent Operators

We define $n_{\mathcal{M}}$ as the maximum number of independent operators of a model $\mathcal{M}$, i.e., the maximum number of operators that can be taken in $\mathcal{M}$ without forming any loop. Necessarily $n_{\mathcal{M}} \leq n$, because all operators can be generated by products of the n spins. Furthermore, the number of spin operators [17] generated by $n_{\mathcal{M}}$ independent operators is $2^{n_{\mathcal{M}}} - 1$, which implies the following relations between $|\mathcal{M}|$ and $n_{\mathcal{M}}$ (see Figure S1):

$$n_{\mathcal{M}} \leq |\mathcal{M}| \leq 2^{n_{\mathcal{M}}} - 1 \qquad \Longleftrightarrow \qquad \log_2(|\mathcal{M}| + 1) \leq n_{\mathcal{M}} \leq \min(n, |\mathcal{M}|) \,. \tag{S.29}$$

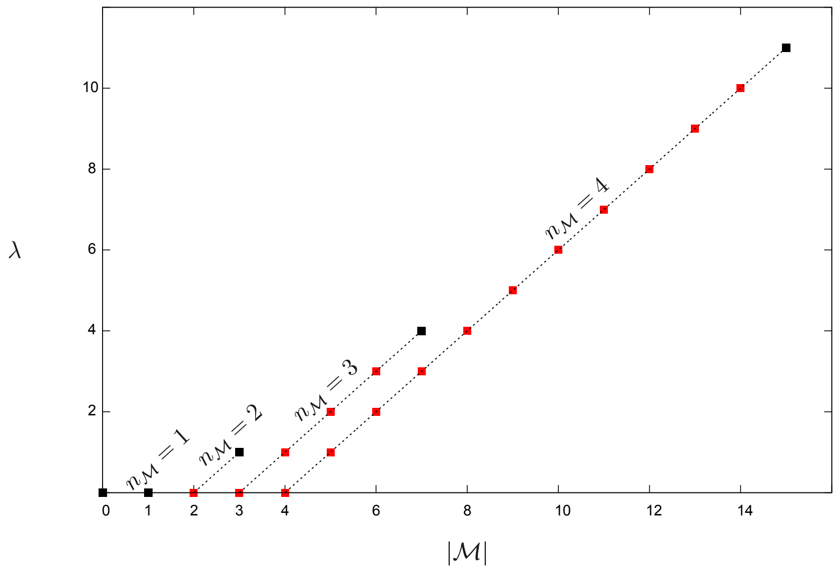

Figure S1. Dependence of λ, defined in SM-3.2, as a function of the number of parameters $|\mathcal{M}|$ for different values of $n_{\mathcal{M}}$. Each value of λ (squares) is extracted from the classification of all the possible models for $n = 4$ (see Table S2 in SM-6). The dash lines corresponds to (S.28) for different values of $n_{\mathcal{M}}$; the black squares, to the value of $\lambda_{\max}$ given in (S.31). Models with $\lambda = 0$ are models with only independent operators, whereas models with $\lambda = \lambda_{\max}$ are equivalent to *sub-complete* models, i.e. complete models on a subset $n_{\mathcal{M}}$ of the n spins.

By definition, $n_{\mathcal{M}}$ is invariant under GT and is thus a property of each complexity class. Table S1, for instance, reports the value of $n_{\mathcal{M}}$ for each class of models with $n = 3$. As an important consequence, any model with $n_{\mathcal{M}} < n$ can be mapped (through a GT) to a model involving only $n_{\mathcal{M}}$ spins. See for instance the class displayed in Figure 1 of the main text: each model involves $|\mathcal{M}| = 7$ operators for $n = 4$ spins, but only $n_{\mathcal{M}} = 3$ are independent; consequently any of these models can be mapped through a GT to a model that involves only 3 spins (see model a) in Figure 1).

SM-3.2. Loop Structure of A Model

For any model $\mathcal{M}$, the corresponding set of loops $\mathcal{L}$ has a finite cardinality [18]. Let us define the *disjunctive union* (or *symmetric difference*) of two loops ℓ_1 and ℓ_2 as the set that contains the operators that occur in ℓ_1 but not in ℓ_2 and *viceversa*: $\ell_1 \oplus \ell_2 = (\ell_1 \cup \ell_2) \backslash (\ell_1 \cap \ell_2)$. The set $\mathcal{L}$ is closed under disjunctive union $\oplus$: indeed if ℓ_1 and ℓ_2 are two elements of $\mathcal{L}$, then $\ell_1 \oplus \ell_2$ is also in $\mathcal{L}$ [19]. We can thus find a *minimal generating set* of loops, of cardinality $\lambda \leq |\mathcal{L}|$, that can generate the whole set $\mathcal{L}$. Finally, we note that the operation $\oplus$ is commutative and that any element of $\mathcal{L}$ is of order 2: for any loop ℓ, $\ell \oplus \ell = \{\emptyset\}$. This way, the total number of loops that can be formed with λ generating loops is $|\mathcal{L}| = 2^\lambda$, including the empty loop $\{\emptyset\}$, and consequently, the total number of non-empty loops of any model is of the form $2^\lambda - 1$.

Mathematically, for any model $\mathcal{M}$, the corresponding set of loop $\mathcal{L}$ forms a finite Abelian group associated with the operator of disjunctive union $\oplus$, the neutral element being the empty loop $\{\emptyset\}$. Each element of this group is of order two, which implies that the cardinality of the group is of the form 2^λ, where λ is then the cardinality of the minimal set of generators of $\mathcal{L}$.

Let us now prove the relation in (S.28). Consider the model $\mathcal{M}$ with $|\mathcal{M}|$ operators, of which at most $n_{\mathcal{M}}$ are independent. Let us take one maximal subset of independent operators of $\mathcal{M}$ and call it $I_{\mathcal{M}}$: by construction, the number of operators in this subset is $|I_{\mathcal{M}}| = n_{\mathcal{M}}$ and the set of loops that can be formed with these operators is necessarily empty. If $|\mathcal{M}| = n_{\mathcal{M}}$, then the set of loops of $\mathcal{M}$ is empty, which is consistent with $\lambda = 0$ in (S.28). If instead $|\mathcal{M}| > n_{\mathcal{M}}$, then, for any other operators $\phi^\nu \in \mathcal{M} \backslash I_{\mathcal{M}}$, we can find a subset $\mathcal{P}^\nu \subseteq I_{\mathcal{M}}$ such that the set $\ell^\nu = \{\phi^\nu\} \cup \mathcal{P}^\nu$ is a loop of $\mathcal{M}$, i.e.,

$$\phi^\nu \times \left(\prod_{\mu \in \mathcal{P}^\nu} \phi^\mu \right) = 1\,. \tag{S.30}$$

This procedure, for each different operator $\phi^\nu \in \mathcal{M} \backslash I_{\mathcal{M}}$, produces a different loop, so the set of $|\mathcal{M}| - n_{\mathcal{M}}$ loops $\ell^\nu = \{\phi^\nu\} \cup \mathcal{P}^\nu$ built in this way is a minimal generating set of $\mathcal{L}_{\mathcal{M}}$. Note indeed that each loop $\ell \in \mathcal{L}_{\mathcal{M}}$ can be decomposed in the loops ℓ^ν in an unique way. Therefore $\lambda = |\mathcal{M}| - n_{\mathcal{M}}$. Table S1, reports the values of $|\mathcal{M}|$, $n_{\mathcal{M}}$ and λ for $n \leq 4$ and (S.28) can be verified for each class of complexity. For a fixed number $n_{\mathcal{M}}$ of independent operators, λ thus grows linearly as a function of the number of parameters of $\mathcal{M}$ up to a maximum value (see Figure S1),

$$\lambda_{\max} = |\Omega_{n_{\mathcal{M}}} \backslash \{1\}| - n_{\mathcal{M}} = 2^{n_{\mathcal{M}}} - 1 - n_{\mathcal{M}}\,, \tag{S.31}$$

where $\Omega_{n_{\mathcal{M}}}$ is the set of operators that can be generated from $n_{\mathcal{M}}$ spins. The value of $\lambda_{\max}$ is associated with the maximum set of loops that can be generated from $n_{\mathcal{M}}$ spins, that corresponds to the class of complexity of *sub-complete models* on $n_{\mathcal{M}}$ spins, i.e., complete models on a subset $n_{\mathcal{M}}$ of the n spins.

SM-3.3. Complexity Classes of Complementary Models

Consider a model $\mathcal{M}$ with $|\mathcal{M}|$ operators, we define the *complementary model* $\mathcal{M}_c$ as the unique model that contains all the operators that are not in $\mathcal{M}$, i.e., $\mathcal{M}_c = \Omega_n \backslash \{1\} \backslash \mathcal{M}$, which can also be written as:

$$\mathcal{M}_c \cup \mathcal{M} = \Omega_n \backslash \{1\} \qquad \text{and} \qquad \mathcal{M}_c \cap \mathcal{M} = \emptyset\,. \tag{S.32}$$

By definition, $\mathcal{M}_c$ has exactly $|\mathcal{M}_c| = 2^n - 1 - |\mathcal{M}|$ operators. Using the properties of gauge transformations, it can be shown that, if two models belong to the same complexity class $\mathcal{C}$, then their respective complementary models also belong to the same class $\mathcal{C}_c$ (see proof below). As a consequence, the two corresponding classes of complexity (named *complementary classes*) have the same cardinality and the number of complexity classes with $|\mathcal{M}|$ parameters is equal to the number of classes with $2^n - 1 - |\mathcal{M}|$ parameters. Observe, for instance, in Table S1 the symmetry in the cardinality of classes of complementary models (starting from the first line and the last line of the table).

Proof. Consider a model $\mathcal{M}$, its complementary model $\mathcal{M}_c$, and their respective classes of complexity, $\mathcal{C}$ and $\mathcal{C}_c$. Let us take a GT $\mathcal{T}$ and define the transformed models $\mathcal{M}' = \mathcal{T}[\mathcal{M}]$ and $\mathcal{M}'_c = \mathcal{T}[\mathcal{M}_c]$. By definition, $\mathcal{M}' \in \mathcal{C}$ and $\mathcal{M}'_c \in \mathcal{C}_c$. As GT are bijections of the space of operators and $\mathcal{M}_c \cap \mathcal{M} = \emptyset$, the new sets of operators obtained after GT are necessarily disjoint: $\mathcal{M}'_c \cap \mathcal{M}' = \emptyset$. Besides, $|\mathcal{M}'_c| + |\mathcal{M}'| = |\mathcal{M}_c| + |\mathcal{M}| = |\Omega_n \backslash \{1\}|$, such that the two models also verify that $\mathcal{M}'_c \cup \mathcal{M}' = \Omega_n \backslash \{1\}$. In other words, $\mathcal{M}'_c$ is the complementary model of $\mathcal{M}'$. We thus obtain that, if two models belong to the same class of complexity $\mathcal{C}$, then their respective complementary models also belong to the same class $\mathcal{C}_c$, thus called *complementary class* of $\mathcal{C}$. □

SM-4. A General Argument for the Calculation of $c_{\mathcal{M}}$

In this section we provide a general argument for the calculation of the complexity $c_{\mathcal{M}}$: in the first part we compute the complexity of models with only independent operators and show that any independent operator (that doesn't enter in a loop) contributes as $\log \pi$ to $c_{\mathcal{M}}$; while in the second part we suggest that this value constitutes an upper bound for the complexity of any operator.

SM-4.1. Models with Only Independent Operators

At fixed number of spins n, every model with $|\mathcal{M}| = n_{\mathcal{M}}$ independent operators and non-degenerated parameters belongs to the same class of complexity (see SM-3), which is the class of models with $\mathcal{L} = \{\{\emptyset\}\}$. The number of models in such a class is

$$\mathcal{N}_n^{\text{ind}}(n_{\mathcal{M}}) = \frac{\prod_{i=0}^{n_{\mathcal{M}}-1}(2^n - 2^i)}{n_{\mathcal{M}}!}\,, \tag{S.33}$$

which is the number of possible ways [20] of choosing $n_{\mathcal{M}}$ independent operators in $\Omega_n \backslash \{1\}$ divided by their permutations. Notice that $1 \leq n_{\mathcal{M}} \leq n$, as $n+1$ operators are necessarily forming at least one loop. In particular, for models with $|\mathcal{M}| = 1$ operator, we recover that $\mathcal{N}_n^{\text{ind}}(1) = |\Omega_n|$ (any operator can be chosen); and, for models with $n_{\mathcal{M}} = n$ independent operators, we obtain that $\mathcal{N}_n^{\text{ind}}(n) = \mathcal{N}_{GT}/n!$. For instance, with $n = 4$, Equation (S.33) gives: $\mathcal{N}_4^{\text{ind}}(2) = 105$ for $n_{\mathcal{M}} = 2$, $\mathcal{N}_4^{\text{ind}}(3) = 420$ and $\mathcal{N}_4^{\text{ind}}(4) = 840$, that match with the results in Table S2.

The partition function of a model with $n_{\mathcal{M}} = |\mathcal{M}|$ independent operators contains only the first term of Equation (7) of the main text, $Z(\boldsymbol{g}) = 2^n \prod_{\mu \in \mathcal{M}} \cosh(g^\mu)$. The Fisher information matrix (FIM), in Equation (5) of the main text, is therefore diagonal and reads $J_{\mu\nu}^{\text{ind}}(\boldsymbol{g}) = \delta_{\mu\nu}[1 - \tanh^2(g^\mu)]$, which finally leads to the complexity term:

$$e^{c_{\mathcal{M}}} = \int_{\mathbb{R}^{|\mathcal{M}|}} \prod_{\mu \in \mathcal{M}} \sqrt{1 - \tanh^2(g^\mu)}\, \mathrm{d}\boldsymbol{g} = \left[\int_{\mathbb{R}} \sqrt{1 - \tanh^2(g)}\, \mathrm{d}g\right]^{|\mathcal{M}|} = \pi^{|\mathcal{M}|}\,. \tag{S.34}$$

For the same reason, any operator of a model, that doesn't enter in any loop of the model, contributes with a term $\log \pi$ to the complexity $c_{\mathcal{M}}$. Indeed, let us consider a model $\mathcal{M}$ with $|\mathcal{M}|$ operators, including K independent $\phi^1, \dots \phi^K$. We can then introduce a model $\mathcal{M}'$ formed by the set of non-independent operators of $\mathcal{M}$, and a model $\mathcal{M}_{\text{ind}} = \{\phi^1, \dots \phi^K\}$, so that $\mathcal{M} = \mathcal{M}' \cup \mathcal{M}_{\text{ind}}$, $|\mathcal{M}| = |\mathcal{M}'| + K$ and $\mathcal{L}_{\mathcal{M}'} = \mathcal{L}_{\mathcal{M}}$. As a consequence the partition function in Equation (7) of the main text can be factorized in the two models:

$$Z_{\mathcal{M}}(\boldsymbol{g}) = 2^n \prod_{\mu \in \mathcal{M}_{\text{ind}}} \cosh(g^\mu) \prod_{\mu \in \mathcal{M}'} \cosh(g^\mu) \left[\sum_{\ell \in \mathcal{L}_{\mathcal{M}'}} \prod_{\mu \in \ell} \tanh(g^\mu) \right] \tag{S.35}$$

$$= \frac{1}{2^n} Z_{\mathcal{M}_{\text{ind}}}(g^1, \dots, g^K)\, Z_{\mathcal{M}'}(g^{K+1}, \dots, g^{\mathcal{M}}) \tag{S.36}$$

Using the previous argument, we obtain that the FIM of $\mathcal{M}$ is a block matrix:

$$\mathbb{J}_{\mathcal{M}}(\boldsymbol{g}) = \begin{pmatrix} \mathbb{J}^{\text{ind}}(g^{[1,K]}) & 0 \\ 0 & \mathbb{J}_{\mathcal{M}'}(\boldsymbol{g}') \end{pmatrix} \tag{S.37}$$

where $\mathbb{J}^{\text{ind}}$ is the diagonal matrix previously introduced (for models with only independent operators). Finally, using the property of the determinant, $\det \mathbb{J} = \det[\mathbb{J}^{\text{ind}}] \det[\mathbb{J}_{\mathcal{M}'}]$, we obtain that

$$e^{c_{\mathcal{M}}} = e^{c_{\mathcal{M}_{\text{ind}}}} \times e^{c_{\mathcal{M}'}} = \pi^K \times e^{c_{\mathcal{M}'}}\,. \tag{S.38}$$

Notice that, in (S.34), the stochastic complexity for $|\mathcal{M}|$ independent operators is linear in the number of operators $|\mathcal{M}|$, which is of the same form than the first penalty term in the BIC (penalty due to the number of operators – see Equation (3) of the main text).

SM-4.2. General Argument

Given a dataset $\hat{\boldsymbol{s}} = (\boldsymbol{s}^{(1)}, ..., \boldsymbol{s}^{(N)})$ where $\boldsymbol{s}^{(i)}$ are n-spins configurations, the maximum likelihood of exponential models defined in (S.7), is achieved when the empirical averages $\hat{\varphi}^\mu$ (S.14) of the operators ϕ^μ match the population averages φ^μ (S.16), and takes the value:

$$P(\hat{\boldsymbol{s}}|\hat{\boldsymbol{g}}, \mathcal{M}) = e^{-NS(\hat{\boldsymbol{\varphi}})},$$

where $S(\hat{\boldsymbol{\varphi}})$ is the entropy of $P(\boldsymbol{s}|\hat{\boldsymbol{g}})$. So we can introduce a delta function enforcing Equation (S.15) in Equation (S.21) for each operator $\mu \in \mathcal{M}$, resulting in

$$\left(\frac{2\pi}{N}\right)^{|\mathcal{M}|/2} \sum_{\hat{\boldsymbol{s}}} P(\hat{\boldsymbol{s}}|\hat{\boldsymbol{g}}, \mathcal{M}) = \left(\frac{2\pi}{N}\right)^{|\mathcal{M}|/2} \int_{-1}^{1} d\boldsymbol{\varphi} e^{-NS(\boldsymbol{\varphi})} \sum_{\hat{\boldsymbol{s}}} \prod_{\mu \in M} \delta\left(\varphi^\mu - \frac{1}{N}\sum_{i=1}^{N} \phi^\mu(\boldsymbol{s}^{(i)})\right). \tag{S.39}$$

The sum over samples is zero unless $\boldsymbol{\varphi}$ can be realized in at least one sample. This means that $\varphi^\mu = (2k^\mu - N)/N$ can only attain $N+1$ values for $k^\mu = 0, 1, \dots, N$. Yet not all values of $\boldsymbol{\varphi}$ can be realized. For instance, if $\phi^1 = s_1$, $\phi^2 = s_2$ and $\phi^3 = s_1 s_2$, when $\hat{\varphi}^1 = \hat{\varphi}^2 = 1$ there are no samples for which $\hat{\varphi}^3 \neq 1$. So let

$$\mathcal{F} = \left\{ \boldsymbol{\varphi}' : \exists \hat{\boldsymbol{s}} \;\; (\varphi')^\mu = \hat{\varphi}^\mu(\hat{\boldsymbol{s}}) \; \forall \mu \in \mathcal{M} \right\} \tag{S.40}$$

be the set of feasible values of $\boldsymbol{\varphi}$, known as the *marginal polytope* [21]. Then the integral in Equation (S.39) becomes a sum

$$\left(\frac{2\pi}{N}\right)^{|\mathcal{M}|/2}\sum_{\hat{s}} P(\hat{s}|\hat{g},\mathcal{M}) = \left(\frac{2\pi}{N}\right)^{|\mathcal{M}|/2}\sum_{\varphi\in\mathcal{F}} Q(\varphi)e^{-NS(\varphi)} \tag{S.41}$$

where $Q(\varphi')$ is the number of samples $\hat{s}$ for which $\hat{\varphi}^\mu(\hat{s}) = (\varphi')^\mu$ for all $\mu \in \mathcal{M}$. $Q(\varphi)$ can be estimated in the weak dependence limit where it is given by

$$Q(\varphi) \simeq \prod_{\mu\in\mathcal{M}} \binom{N}{N\frac{1+\varphi^\mu}{2}} \simeq \left(\frac{2}{\pi N}\right)^{|\mathcal{M}|/2} e^{NS(\varphi)} \prod_{\mu\in\mathcal{M}} \left[1-(\varphi^\mu)^2\right]^{-1/2}.$$

so

$$\left(\tfrac{2\pi}{N}\right)^{|\mathcal{M}|/2}\textstyle\sum_{\hat{s}} P(\hat{s}|\hat{g},\mathcal{M}) \simeq \left(\tfrac{2}{N}\right)^{|\mathcal{M}|}\sum_{\varphi\in\mathcal{F}}\prod_{\mu\in\mathcal{M}}\left[1-(\varphi^\mu)^2\right]^{-1/2} \simeq \int_{\mathcal{F}} d\varphi \prod_{\mu\in\mathcal{M}}\left[1-(\varphi^\mu)^2\right]^{-1/2} \tag{S.42}$$

where we have turned the sum over φ^μ into an integral, observing that $d\varphi^\mu = 2/N$. In the limit $N\to\infty$ we finally get

$$e^{c_{\mathcal{M}}} = \int dg\sqrt{\det J(g)} \simeq \int_{\mathcal{F}} d\varphi \prod_{\mu\in\mathcal{M}}\left[1-(\varphi^\mu)^2\right]^{-1/2}. \tag{S.43}$$

This is a quite interesting result. It tells us that the complexity of a model is related to how operators of the model constrain the values that the expected values of other operators can take. All integrals in Equation (S.43) are over a subset $\mathcal{F}$ of the hypercube $[-1,1]^{|\mathcal{M}|}$, with the same integrand. Then the complexity uniquely depends on the volume of $\mathcal{F}$ under the measure $p(\varphi) \propto \prod_{\mu\in\mathcal{M}}(1-(\varphi^\mu)^2)^{-1/2}$.

The approximation used becomes exact in the case of independent operators and is likely an upper bound otherwise.

As a corollary, we find that the most complex models are those where all operators are independent and $\mathcal{F} = [-1,1]^{|\mathcal{M}|}$. In this case the integral takes the value $\pi^{|\mathcal{M}|}$ (see SM-4.1). The least complex models, instead, are those where operators constrain themselves as much as possible. This correspond to models where all operators depend on the same subset of spins.

SM-5. The Complexity of Complete Models

In this section we derive an analytic expression for the complexity of *complete models* exploiting the invariance under reparametrization of Jeffreys prior [10] distribution over the parameters.

SM-5.1. Properties of the Complete Model

The *complete model* $\overline{\mathcal{M}}$ involves all the 2^n-1 operators of $\Omega_n\backslash\{1\}$. This model presents the peculiarity that the number of parameters $|\overline{\mathcal{M}}|$ equals the number of independent parameters that are needed to specify a generic distribution $p(s)$ on the spin configurations. In order to make this more explicit let us label by an integer $i \in \{0,1,\ldots,2^n-1\}$ all configurations $s^i \in \mathcal{S}$ and let $p^i = p(s^i)$. We can take $p^0 = p(s^0)$ to be constrained by normalisation: $\sum_{i=0}^{2^n-1} p^i = 1$, which leaves $2^n-1 = |\overline{\mathcal{M}}|$ free parameters p^i, $i \in \{1,\ldots,2^n-1\}$. Re-writing (S.7) with this notation,

$$p^i = \exp\left[\sum_{\mu=1}^{2^n-1} g^\mu\,\phi^\mu(s^i) - \log Z_{\mathcal{M}}(g)\right], \tag{S.44}$$

taking the log, multiplying by $\phi^\nu(s^i)$ and summing over i with the relation in (S.3), leads to

$$g^{\nu} = \frac{1}{2^n} \sum_{i=0}^{2^n-1} \phi^{\nu}(\boldsymbol{s}^i) \log p^i, \qquad \text{where} \;\; p^0 = 1 - \sum_{i=1}^{2^n-1} p^i \,. \tag{S.45}$$

We call this model *complete* in the sense that (S.44) and (S.45) define a bijection between the sets of parameters $\boldsymbol{g} = \{g^{\mu}\}_{\mu \in \{1,\ldots,2^n-1\}}$ and the sets of $2^n - 1$ probabilities $\boldsymbol{p} = \{p^i\}_{i \in \{1,\ldots,2^n-1\}}$, provided that one includes the values $\pm\infty$ as legitimate values for the individual parameters g^{μ}. This shows that this basis of models is *complete* in the sense that any probability distribution can be represented within this class of models.

SM-5.2. Complexity of the Complete Model

The bijection between parameters $\{g^{\mu}\}$ and probabilities $\{p^i\}$ indicates that $\boldsymbol{p}$ constitutes a suitable parametrization for the *complete model*. The Fisher Information matrix in $\boldsymbol{p}$ reads:

$$J_{ij}(\boldsymbol{p}) = -\left\langle \partial_{p^i}\partial_{p^j} \log P(\boldsymbol{s}\,|\,\boldsymbol{p},\mathcal{M}) \right\rangle_P = \frac{\delta_{i,j}}{p^i} + \frac{1}{p^0}\,, \qquad \text{for all } (i,j) \in \{1,\ldots,2^n-1\}^2\,. \tag{S.46}$$

Using that the *volume element*

$$\mathrm{d}V = \sqrt{\det \mathbb{J}(\boldsymbol{g})}\; \mathrm{d}\boldsymbol{g} = \sqrt{\det \mathbb{J}(\boldsymbol{p})}\; \mathrm{d}\boldsymbol{p}\,, \tag{S.47}$$

is invariant under re-parametrisation [22], we can express the complexity in the new set of parameters $\boldsymbol{p}$:

$$c_{\mathcal{M}} = \log \int \mathrm{d}\boldsymbol{p}\, \sqrt{\det \mathbb{J}(\boldsymbol{p})}\,. \tag{S.48}$$

In the p-parameters, the determinant of $\mathbb{J}(\boldsymbol{p})$ can be more easily worked out by rewriting the FIM as:

$$\mathbb{J}(\boldsymbol{p}) = \mathbb{D}\left(\mathbb{1} + \mathbf{v}\mathbf{w}^t\right) \tag{S.49}$$

where $\mathbb{D}$ is a diagonal matrix with entries $D_{ii} = 1/p_i$, $\mathbb{1}$ is the identity matrix, and, $\mathbf{v}$ and $\mathbf{w}$ are two vectors with elements

$$v_i = p^i \quad \text{and} \quad w_j = \frac{1}{p^0}\,. \tag{S.50}$$

Finally by using the properties of the determinant, $\det(\mathbb{D}\left(\mathbb{1} + \mathbf{v}\mathbf{w}^t\right)) = \det\mathbb{D}\,\det(\mathbb{1} + \mathbf{v}\mathbf{w}^t)$ and $\det(\mathbb{1} + \mathbf{v}\mathbf{w}^t) = 1 + \mathbf{v}^t\mathbf{w}$, one gets

$$\det \mathbb{J}(\boldsymbol{p}) = \prod_{i=0}^{2^n-1} \frac{1}{p^i}\,. \tag{S.51}$$

So the complexity of the complete model is

$$c_{\overline{\mathcal{M}}} = \log \int_{[0,1]^{2^n}} \mathrm{d}\boldsymbol{p}\, \delta\left(\sum_{i=0}^{2^n-1} p^i - 1\right) \prod_{i=0}^{2^n-1} \frac{1}{\sqrt{p^i}} = 2^{n-1} \log \pi - \log \Gamma(2^{n-1}) \tag{S.52}$$

SM-6. Complexity: Numerical Estimates

In this Section we deal with the computation of the complexity penalty $c_{\mathcal{M}}$, defined in Equation (5) of the main text. We start by considering the generic model with an arbitrary loop structure and we derive the expression of the complexity integral for a model with a single loop. Finally we assess numerically the complexity for all the models on systems of $n \leq 4$ spins. Here we will focus on models with non-degenerated parameters, leaving the discussion on degeneracy to SM-7.

SM-6.1. Generic Model

In this section we will derive an expression for the complexity integral of a generic model suitable for numerical integration. Since the contribution to the complexity of independent operators has been derived in SM-4, here we will focus on models in which there are no independent operators, i.e., every operator participates in at least into one loop.

The elements of the FIM in Equation (5) of the main text, obtained by taking the derivatives of the logarithm of the partition function Equation (7) of the main text, are for a generic model $\mathcal{M}$:

$$J_{\mu\nu} = \begin{cases} \left(1-\gamma_\mu^2\right)\left(1 - 2\frac{\chi_\mu}{1+\chi} - \frac{1-\gamma_\mu^2}{\gamma_\mu^2}\frac{\chi_\mu^2}{(1+\chi)^2}\right) & \text{for} \quad \mu = \nu \\ \frac{1-\gamma_\mu^2}{\gamma_\mu(1+\chi)}\frac{1-\gamma_\nu^2}{\gamma_\nu(1+\chi)}\left(\chi_{\mu,\nu}(1+\chi) - \chi_\mu\chi_\nu\right) & \text{for} \quad \mu \neq \nu \end{cases} \tag{S.53}$$

where we have defined

$$\gamma_\mu = \tanh g^\mu \tag{S.54a}$$

$$\chi = \sum_{\ell \in \mathcal{L}_{\mathcal{M}} \setminus \{\emptyset\}} \prod_{\mu \in \ell} \gamma_\mu \tag{S.54b}$$

$$\chi_\mu = \sum_{\ell|\mu} \prod_{\nu \in \ell} \gamma_\nu \tag{S.54c}$$

$$\chi_{\nu,\mu} = \sum_{\ell|\{\nu,\mu\}} \prod_{\sigma \in \ell} \gamma_\sigma \tag{S.54d}$$

and "$\ell|\mu$" ("$\ell|\nu,\mu$") refers to the loops in $\mathcal{L}_{\mathcal{M}}$ in which g^μ (g^μ and g^ν) enters. In light of (S.53) the FIM can be expressed as

$$\mathbb{J}(\boldsymbol{g}) = \mathbb{A}(\boldsymbol{g}) + \mathbb{W}(\boldsymbol{g}) \tag{S.55}$$

where $\mathbb{A}(\boldsymbol{g})$ is a diagonal matrix with $A_{\mu\mu} = J_{\mu\mu} - W_{\mu\mu}$ and $\mathbb{W}(\boldsymbol{g})$ is defined as:

$$W_{\mu\nu} = \left(\frac{1-\gamma_\mu^2}{\gamma_\mu}\right)\frac{\chi_\mu}{1+\chi}\left(\frac{1-\gamma_\nu^2}{\gamma_\nu}\right)\frac{\chi_\nu}{1+\chi}\left(\frac{\chi_{\mu,\nu}}{\chi_\mu\chi_\nu}(1+\chi) - 1\right). \tag{S.56}$$

Splitting $\mathbb{J}(\boldsymbol{g})$ as in (S.55) allows us to rewrite the determinant of the FIM

$$\det \mathbb{J}(\boldsymbol{g}) = \det(\mathbb{A}(\boldsymbol{g}) + \mathbb{W}(\boldsymbol{g})) = \det \mathbb{A}(\boldsymbol{g}) \det(\mathbb{1} + \mathbb{A}^{-1}(\boldsymbol{g})\mathbb{W}(\boldsymbol{g})) \tag{S.57}$$

and to exploit the fact that $\det \mathbb{A}(\boldsymbol{g})$ is simply the product of the diagonal entries of $\mathbb{A}$

$$\det \mathbb{A}(\boldsymbol{g}) = \prod_\mu \left(1-\gamma_\mu^2\right)\left(1 - \frac{1+\gamma_\mu^2}{\gamma_\mu^2}\frac{\chi_\mu}{1+\chi}\right) \tag{S.58}$$

and that $\mathbb{A}^{-1}(\boldsymbol{g})$ is a diagonal matrix with entries ${A^{-1}}_{\mu\mu} = (A_{\mu\mu})^{-1}$. Notice that $\mathbb{1}$ in (S.57) is the identity matrix. Substituting (S.58) in (S.57) one gets:

$$\det \mathbb{J}(g) = \prod_{\mu}\left(1-\gamma_{\mu}^{2}\right)\left[\prod_{\mu}\left(1-\frac{\chi_{\mu}}{1+\chi}\frac{1+\gamma_{\mu}^{2}}{\gamma_{\mu}^{2}}\right)\right](\det(\mathbb{1}+\mathbb{B}(g)))$$
$$B_{\mu\nu} = \frac{\gamma_{\mu}}{\gamma_{\nu}}\frac{1-\gamma_{\nu}^{2}}{1+\chi}\frac{\chi_{\mu,\nu}(1+\chi)-\chi_{\mu}\chi_{\nu}}{(\gamma_{\mu}^{2}(1+\chi)-(1+\gamma_{\mu}^{2})\chi_{\mu})} \tag{S.59}$$

where we have defined the matrix $\mathbb{B}(g) = \mathbb{A}^{-1}(g)\mathbb{W}(g)$. Replacing the determinant of the FIM (Equation (S.59)) in the complexity integral (Equation (5) in the main text) and performing the change of variables $g^{\mu} \to \gamma_{\mu}$, defined in (S.54), yields

$$e^{c_{\mathcal{M}}} = \pi^{|\mathcal{M}|}\int_{[-1,1]^{|\mathcal{M}|}} d\gamma\, q(\gamma)\left[\prod_{\mu\in\mathcal{M}}\sqrt{\left(1-\frac{\chi_{\mu}}{1+\chi}\frac{1+\gamma_{\mu}^{2}}{\gamma_{\mu}^{2}}\right)}\right]\sqrt{\det(\mathbb{1}+\mathbb{B})} \tag{S.60}$$

where

$$q(\gamma) = \frac{1}{\pi^{|\mathcal{M}|}}\prod_{\mu}\left(1-\gamma_{\mu}^{2}\right)^{-1/2}. \tag{S.61}$$

Now (S.60) is prone for standard Monte Carlo integration by random sampling γ according to the pdf $q(\gamma)$ on its bounded support. As one could easily check, $q(\gamma)$ is the measure induced by a set of $|\mathcal{M}|$ independent operators on the hypercube $[-1,1]^{|\mathcal{M}|}$ (see SM-4).

SM-6.2. Models with A Single Loop

We consider models with all their operators involved in a single loop. The contribution to the complexity $c_{\mathcal{M}}$ of any supplementary independent operator (not involved in the loop) was studied in SM-4 (contribution of $\log \pi$ for each supplementary operator), and will not be considered here. Single loop models $\mathcal{M}$ are such that $\mathcal{L} = \{\{\emptyset\},\ell\}$, where $\ell = \{\phi^{1},\ldots,\phi^{|\mathcal{M}|}\} = \mathcal{M}$. All such models with fixed number of operators belong to the same class of complexity. The cardinality of this class can be assessed thinking at the single loop model with $|\mathcal{M}|$ operators as a set of $|\mathcal{M}|-1$ independent operators plus an operator being the product of the independent ones. Following this reasoning the number of single loop models on a n spins system is:

$$\mathcal{N}_{1\,\mathrm{loop}} = \prod_{l=0}^{|\mathcal{M}|-2}\frac{(2^{n}-2^{l})}{|\mathcal{M}|!}, \tag{S.62}$$

where the previous formula was derived in full analogy with the number of models with only independent operators (see SM-4).

Complexity of Single Loop Models

Expression (S.60) for the complexity integral can be notably simplified in case of single loop models. The single loop ℓ of length $|\mathcal{M}|$ reduces (S.54) to $\chi_{\mu,\nu} = \chi_{\mu} = \chi$ and $\chi = \prod_{\mu\in\ell}\gamma_{\mu}$. Enforcing these relations into the determinant of the FIM (S.59) yields

$$\det \mathbb{J}(g) = \prod_{\mu\in\ell}\left(1-\gamma_{\mu}^{2}\right)\left[\prod_{\mu\in\ell}\left(1-\frac{\chi}{1+\chi}\frac{1+\gamma_{\mu}^{2}}{\gamma_{\mu}^{2}}\right)\right](\det(\mathbb{1}+\mathbb{B}))$$
$$B_{\mu\nu} = \frac{\gamma_{\mu}}{\gamma_{\nu}}\frac{1-\gamma_{\nu}^{2}}{1+\chi}\frac{\chi}{(\gamma_{\mu}^{2}-\chi)} \tag{S.63}$$

The matrix $\mathbb{B}(g)$ in (S.63) can be rewritten as the outer product of two vectors:

$$\mathbb{B}(g) = \mathbf{b}\mathbf{c}^t \qquad \text{with} \qquad b_\mu = \frac{\chi}{1+\chi}\frac{\gamma_\mu}{\gamma_\mu^2 - \chi} \quad \text{and} \quad c_\mu = \frac{\alpha_\mu(1-\gamma_\mu^2)}{\gamma_\mu}\,,\ \mu \in \ell \tag{S.64}$$

such that

$$\det(\mathbb{1} + \mathbb{B}) \;=\; 1 + \mathbf{c}^t\mathbf{b} \;=\; 1 + \frac{\chi}{1+\chi}\sum_{\mu\in\ell}\frac{1-\gamma_\mu^2}{(\gamma^2 - \chi)}. \tag{S.65}$$

The resulting expression for the complexity, obtained by replacing (S.65) and (S.63) in Equation (5) of the main text,

$$e^{c_\ell} = \pi^{|\mathcal{M}|}\int_{[-1,1]^{|\mathcal{M}|}} \mathrm{d}\gamma\, q(\gamma)\left[\prod_\mu \sqrt{\left(1 - \frac{\chi}{1+\chi}\frac{1+\gamma_\mu^2}{\gamma_\mu^2}\right)}\,\right]\sqrt{1 + \frac{\chi}{1+\chi}\sum_{\mu\in\ell}\frac{1-\gamma_\mu^2}{(\gamma_\mu^2 - \chi)}}\,, \tag{S.66}$$

is then suited for numerical integration using Monte Carlo methods as explained in section SM-6.1.

Figure 3 of the main text displays the complexity of models with a fixed number $|\mathcal{M}|$ of operators and a single loop ℓ of length k, for different values of k. Such a model has $|\mathcal{M}| - k$ *free* operators (operators not involved in any loop). It can be, for instance, a model with $(|\mathcal{M}| - 1)$ fields (considering that the number of spins $n \geq |\mathcal{M}| - 1$) and one $(k-1)$-body interaction, or a model formed with a closed chain of k pairwise interactions and $|\mathcal{M}| - k$ free fields. The complexity of such a model is $c_\mathcal{M}(k) = c_\ell(k) + (|\mathcal{M}| - k)\log\pi$, where $(|\mathcal{M}| - k)\log\pi$ is the contribution of the free operators and $c_\ell(k)$ is the one of the single loop of length k. For $k = 3$, this latter complexity can be obtained analytically from (S.66),

$$c_\ell(3) = \log\int_{(-1,1)^3}\frac{1}{(1+xyz)^2}\,\mathrm{d}x\mathrm{d}y\mathrm{d}z = \log(\pi^2)\,, \tag{S.67}$$

or directly by setting $n = 2$ in (S.52) (as the complete model for $n = 2$ spins is a single loop of length $k = 3$). We thus obtain that $c_\mathcal{M}(3) = (|\mathcal{M}| - 1)\log\pi$. For larger values of k, the complexity $c_\ell(k)$ is obtained numerically by integrating (S.66) with a Monte Carlo method. Figure 3 of the main text shows that the complexity $c_\mathcal{M}(k)$ of such models increases with the length k of the loop, and saturates for large k at $c_\mathcal{M}(k) \to c_\mathcal{M}(3) + \log\pi = |\mathcal{M}|\log\pi$, which corresponds to the complexity of a model with $|\mathcal{M}|$ independent operators. This can be re-written in terms of the complexity of the single loop: the complexity of the single loop increases with the length k of the loop, starting from $c_\ell(3) = 2\log\pi$ to finally grow for large k as $c_\ell(k) \to k\log\pi$, as if the k operators of the loop were independent.

SM-6.3. All Models for $n \leq 4$

In Table S1 and Table S2, we classified all the models for, respectively, $n = 3$ and $n = 4$.

In the 4-spin system, there are $2^{15} = 32768$ distinct non-degenerate models. We counted 20160 possible gauge transformations, which is in agreement with Equation (S.25). By applying all gauge transformations to all models, we find that models can be classified in 46 complexity classes (see Table S2). The number of different values of complexity $c_\mathcal{M}$ to be estimated numerically with Equation (S.66) is thus drastically reduced, from 32768 to only 46. In the 3-spin system, there are $2^7 = 128$ non-degenerate models spread over 10 complexity classes (see Table S1). Note that the 3-spin system is a sub-case of a 4-spin system (see all classes with $n_\mathcal{M} \leq 3$ in Table S2): every complexity class of the 3-spin system is also a class of the 4-spin system, with all characteristics preserved, except for the number of elements in the class (which takes into account the additional spin).

The comprehensive study of classes for $n = 3$ and $n = 4$ allows comparing with the results of the previous sections. First, the relation between $|\mathcal{M}|$, λ and $n_{\mathcal{M}}$ in (S.28) is always verified for each class of the two tables. We can also observe, in both tables, the "symmetry" in the cardinality between classes of models with $|\mathcal{M}|$ operators and their respective complementary classes of models with $2^n - 1 - |\mathcal{M}|$ operators (see SM-3.3). Equation (S.33) for the cardinality of classes with only independent operators (such that $|\mathcal{M}| = n_{\mathcal{M}}$) is also verified here. Finally, let us remark, in Table S1, that two different classes, with different structures (even different number of operators), may have the same value of $c_{\mathcal{M}}$. For instance, the model $\mathcal{M} = \{s_1, s_2, s_3\}$ (in the class of the 4th row) and the model $\mathcal{M} = \{s_1, s_2, s_3, s_2s_3\}$ (class in the 7th row) have both $c_{\mathcal{M}} = 3\log\pi$ even though their structure are clearly different.

Remarkably, the complexity of models is not monotonic in the number $|\mathcal{M}|$ of operators, as it can be verified in Table S1 for $n = 3$ and in Figure 4 in the main text for $n = 4$. Observe for instance in Table S1 that the complete model (with $|\mathcal{M}| = 7$) is much less complex then any model with $|\mathcal{M}| = 6$ operators. At equal number of operators $|\mathcal{M}|$, the maximum of the complexity is achieved by models with only independent operators (see SM-4.2 and Figure 4) in the main text; on the other hand, *sub-complete models*, i.e. models that contain a *complete* model (see SM-5) on a subset of spins, are the simplest. We also notice that complexity decreases when turning an independent interaction into an operator that enters a loop (compare for instance the two complexity classes with $|\mathcal{M}| = 3$ operators in Table S1). In summary, we found that, adding a new operator to a model:

- increases its complexity $c_{\mathcal{M}}$ by $\log\pi$ if this new operator is independent (doesn't enter in any loop)
- increases its complexity by a quantity between 0 and $\log\pi$ if this new operator enter in a single loop; 0 if the length of the loop is $|\ell| = 3$, and then growing values for larger loop length (see SM-6.2).
- if the new operator enter in several loops, the complexity may increase (from always less than $\log\pi$) or decrease; although it is no trivial to predict what will happen, we observe that, at fix number of operators $|\mathcal{M}|$, the closest the model is to a *sub-complete model*, the less complex it is.

Table S1. Summary table of all non-degenerate models of a 3-spin system; models are partitioned in 10 classes. Each line gives the characteristics of one class: the common structure of the models of the class (number of operators $|\mathcal{M}|$, number of independent operators $n_{\mathcal{M}}$, $\lambda = \log_2|\mathcal{L}|$, and lengths $|\ell|$ of each loop $\ell \in \mathcal{L}$), the number of models in the class, and finally, the corresponding value of the complexity $c_{\mathcal{M}}$. The notation a^b means that the element a is repeated b times. The last row corresponds to the *complete* 3-spin model.

$\lvert\mathcal{M}\rvert$	$n_{\mathcal{M}}$	λ	$\{\lvert\ell\rvert, \forall\, \ell \in \mathcal{L}\}$	number of models	$\exp(c_{\mathcal{M}})$
0	0	0	$\{0\}$	1	-
1	1	0	$\{0\}$	7	$\pi \simeq 3.141$
2	2	0	$\{0\}$	21	$\pi^2 \simeq 9.869$
3	3	0	$\{0\}$	28	$\pi^3 \simeq 31.006$
3	2	1	$\{0,3\}$	7	$\pi^2 \simeq 9.869$
4	3	1	$\{0,4\}$	7	$\pi^{3.56831} \simeq 59.427$
4	3	1	$\{0,3\}$	28	$\pi^3 \simeq 31.006$
5	3	2	$\{0,3^2,4\}$	21	$\pi^{3.18346} \simeq 38.252$
6	3	3	$\{0,3^4,4^3\}$	7	$\pi^{3.34058} \simeq 45.790$
**7**	**3**	**4**	$\{\mathbf{0,7,4^7,3^7}\}$	**1**	$\pi^{\mathbf{2.43472}} \simeq \mathbf{16.233}$

Table S2. Summary table of all non-degenerate models of a 4-spin system; models are partitioned in 46 classes. Each line corresponds to one or more classes with the same number of operators $|\mathcal{M}|$ and gives, for each class, the following characteristics: number of independent operators $n_{\mathcal{M}}$, cardinality of the minimal generating set of loops $\lambda = \log_2 |\mathcal{L}|$, and number of models in the class (column "cardinality"). The notation a^b means that the element a is repeated b times. The complexity class taken as an example in Figure 1 and Figure 4 Left is highlighted in bold in this table ($|\mathcal{M}| = 7$ interactions, $\lambda = 4$ and a cardinality of 15).

$\|\mathcal{M}\|$	number of classes	$n_{\mathcal{M}}$	λ	cardinality of each class
0	1	$\{0\}$	$\{0\}$	$\{1\}$
1	1	$\{1\}$	$\{0\}$	$\{15\}$
2	1	$\{2\}$	$\{0\}$	$\{105\}$
3	2	$\{3,2\}$	$\{0,1\}$	$\{420,35\}$
4	3	$\{4,3^2\}$	$\{0,1^2\}$	$\{840,420,105\}$
5	4	$\{4^3,3\}$	$\{1^3,2\}$	$\{1680,840,168,315\}$
6	5	$\{4^4,3\}$	$\{2^4,3\}$	$\{2520,420,1680,280,105\}$
**7**	6	$\{4^5,\mathbf{3}\}$	$\{3^5,\mathbf{4}\}$	$\{840,120,2520,2520,420,\mathbf{15}\}$
8	6	$\{4^6\}$	$\{4^6\}$	$\{840,120,2520,2520,420,15\}$
9	5	$\{4^5\}$	$\{5^5\}$	$\{2520,420,1680,280,105\}$
10	4	$\{4^4\}$	$\{6^4\}$	$\{1680,840,168,315\}$
11	3	$\{4^3\}$	$\{7^3\}$	$\{840,420,105\}$
12	2	$\{4^2\}$	$\{8^2\}$	$\{420,35\}$
13	1	$\{4\}$	$\{9\}$	$\{105\}$
14	1	$\{4\}$	$\{10\}$	$\{15\}$
15	1	$\{4\}$	$\{11\}$	$\{1\}$

To conclude, our close analysis of the $n = 4$ spin case suggests that the simplest models are those where operators concentrate their support on a subset of spins (and their equivalent models), as opposite to "sparse" models with many independent parameters. More precisely, for fixed value of $|\mathcal{M}|$, classes with lower value of $n_{\mathcal{M}}$ (i.e., with less independent operators) are less complex (see colors in Figure 4 in the main text). They are the classes that contain at least one model whose interactions are grouped on a subset $n_{\mathcal{M}}$ of the n spins. Finally, among these models, the least complex are the ones equivalent to the model that is as close as possible to a *sub-complete model* (exactly a sub-complete model for $|\mathcal{M}| = 2^{n_{\mathcal{M}}} - 1$, see $|\mathcal{M}| = 1, 3, 7$ and 15 in Figure 4 in the main text).

SM-7. Degenerate Models

In degenerate models at least two operators, say ϕ^μ and ϕ^ν, have the same parameter, i.e., $g^\mu = g^\nu$ (see SM-0.3). Since the mapping between parameters and operators is no longer bijective, for a degenerate model $\mathcal{M}$, together with the $|\mathcal{M}|$ operators $\boldsymbol{\phi}$ and m parameters $\boldsymbol{g}$, one requires to specify the matrix $\mathbb{U}$ (S.10), that maps operators into parameters [23]. The degeneracy coefficient $\alpha_i = \sum_j U_{ij}$ defines the number of operators parametrized by g^i, implying that, if the number of parameters is m, while the number of operators is $|\mathcal{M}|$, then $\sum_{i=1}^m \alpha_i = |\mathcal{M}|$.

The partition function (see SM-2) of a generic degenerate spin model $\mathcal{M}$, parametrized by $\boldsymbol{g}$ is

$$Z_{\mathcal{M}}(\boldsymbol{g}) = 2^n \prod_{j=1}^{m} (\cosh g^j)^{\alpha_j} \sum_{\ell \in \mathcal{L}_{\mathcal{M}}} \prod_{i \in \ell} (\tanh g^i)^{\beta_i(\ell)} \,. \tag{S.68}$$

This expression extends Equation (7) of the main text to the case of degenerated parameters. Here $i \in \ell$ means that there is at least one operator ϕ^μ, parametrized by g^i, such that ϕ^μ enters the loop ℓ ($\mu \in \ell$ following the notation of SM-1.2). Finally $\beta_i(\ell)$ [24] denotes the degeneracy coefficient of g^i in loop ℓ (how many operators parametrized by g^i enter the loop ℓ).

SM-7.1. Independent Operators and Parameters

The partition function of a model with $n_{\mathcal{M}} = |\mathcal{M}|$ independent operators and m parameters with degeneracy coefficients $(\alpha_1, \ldots, \alpha_m)$ contains only the first term of (S.68), $Z_{\mathcal{M}}(\boldsymbol{g}) = 2^n \prod_{i=1}^{m} \cosh(g^i)^{\alpha_i}$. As a consequence its complexity is:

$$e^{c_{\mathcal{M}}} = \pi^m \prod_{i=1}^{m} \sqrt{\alpha_i}. \tag{S.69}$$

such that any parameter g^i contributes with a term $\log(\sqrt{\alpha_i}\pi)$ to the complexity $c_{\mathcal{M}}$.

Consider now a generic degenerate model $\mathcal{M}$ with $|\mathcal{M}|$ operators, including K independent $\phi^1, \ldots \phi^K$. We can then introduce a model $\mathcal{M}'$ formed by the set of non-independent operators of $\mathcal{M}$ (and relative parameters), and a model $\mathcal{M}_{\text{ind}} = \{\phi^1, \ldots \phi^K\}$ (and relative parameters). Suppose now that the set of parameters of the two models $\mathcal{M}'$ and $\mathcal{M}_{\text{ind}}$ is disjoint, meaning that there is no parameter parametrizing both operators in $\mathcal{M}'$ and $\mathcal{M}_{\text{ind}}$. It follows that $\mathcal{M} = \mathcal{M}' \cup \mathcal{M}_{\text{ind}}$, such that, analogously to the non-degenerated case (see SM-4.1), the partition function (S.68) can be factorized in the two models, and so does the complexity:

$$e^{c_{\mathcal{M}}} = e^{c_{\mathcal{M}_{\text{ind}}}} \times e^{c_{\mathcal{M}'}} = \pi^{m^{\text{ind}}} \prod_{i=1}^{m^{\text{ind}}} \sqrt{\alpha_i} \times e^{c_{\mathcal{M}'}} . \tag{S.70}$$

where m^{ind} is the number of parameters in $\mathcal{M}_{\text{ind}}$ (that possibly differs from the number of operators in $\mathcal{M}_{\text{ind}}$, K).

Notice that if the sets of operators of $\mathcal{M}'$ and $\mathcal{M}_{\text{ind}}$ are disjoint while the sets of parameters aren't, the complexity doesn't factorize in the two models. By comparing this result with the non degenerate case (see SM-4) degenerating parameters reduces the number of independent operators of a model, decreasing the complexity of the model itself. For models with only independent parameters this statement can be easily checked, as the complexity of a model with $|\mathcal{M}|$ independent operators and $|\mathcal{M}|$ parameters is larger than the complexity of a model $|\mathcal{M}|$ independent operators and $m \leq |\mathcal{M}|$ parameters, since $\pi^{|\mathcal{M}|} \leq \pi^m \prod_{i=1}^{m} \sqrt{\alpha_i}$ if $\sum_{i=1}^{m} \alpha_i = |\mathcal{M}|$. The fact that degeneracy of parameters reduces the complexity of a model holds also in loopy models, as we show in section SM-7.3.

Complexity Classes

In case of degenerated parameters the complexity of models with $n_{\mathcal{M}} = |\mathcal{M}|$ independent operators depends only on the number of parameters and the degeneracy coefficients α_i, as shown in SM-7.1. Specifically each class of complexity is identified by the number of parameters $m \leq |\mathcal{M}|$, the set of degeneracy coefficients and their multiplicities $\{(\alpha_{j_1}, r_{j_1}), ..., (\alpha_{j_K}, r_{j_K})\}$, with $\alpha_{j_1} < ... < \alpha_{j_K}$ and K total number of distinct values that the degeneracy coefficients α_i take, such that $\sum_{i=1}^{K} \alpha_{j_i} r_{j_i} = |\mathcal{M}|$ and $\sum_{i=1}^{K} r_{j_i} = m$. Then the cardinality of such a class of complexity is

$$\mathcal{N}_{\text{ind}}^{deg} = \mathcal{N}_{\text{ind}} \frac{n_{\mathcal{M}}!}{(\alpha_{j_1}!)^{r_{j_1}} \cdot ... \cdot (\alpha_{j_K}!)^{r_{j_K}}} \frac{1}{r_{j_1}! \cdot ... \cdot r_{j_K}!} \tag{S.71}$$

the number of possible ways of choosing $n_{\mathcal{M}}$ independent operators $\mathcal{N}_{\text{ind}}$ (S.33) times the number of partitions of a set of $n_{\mathcal{M}}$ elements in exactly m subsets of which r_{j_1} of cardinality α_{j_1}, ..., and r_{j_K} of cardinality α_{j_K}.

SM-7.2. Generic Model

The derivation of the complexity for the generic model in case of non-degenerated parameters (see SM-6.1) can be straightforwardly extended to the case of degenerate models. In particular the FIM (hessian of logarithm of the partion function (S.68)) is

$$J_{ij} = \begin{cases} \left(1-\gamma_i^2\right)\left(\alpha_i + \frac{(1-\gamma_i^2)}{\gamma_i^2(\chi+1)}\left[\chi_{i,i}-\chi_i\right] - 2\frac{\chi_i}{1+\chi} - \frac{1-\gamma_i^2}{\gamma_i^2}\frac{\chi_i^2}{(1+\chi)^2}\right) & \text{for} \quad i=j \\ \frac{1-\gamma_i^2}{\gamma_i(1+\chi)}\frac{1-\gamma_j^2}{\gamma_j(1+\chi)}\left(\chi_{i,j}(1+\chi)-\chi_i\chi_j\right) & \text{for} \quad i\neq j \end{cases} \tag{S.72}$$

where

$$\gamma_i = \tanh g^i \tag{S.73a}$$

$$\chi = \sum_{\ell\in\mathcal{L}_\mathcal{M}\setminus\{\emptyset\}} \prod_{i\in\ell} \gamma_i^{\beta_i(\ell)} \tag{S.73b}$$

$$\chi_i = \sum_{\ell|i} \beta_i(\ell) \prod_{j\in\ell} \gamma_j^{\beta_j(\ell)} \tag{S.73c}$$

$$\chi_{i,j} = \sum_{\ell|\{i,j\}} \beta_i(\ell)\beta_j(\ell) \prod_{m\in\ell} \gamma_m^{\beta_m(\ell)} \tag{S.73d}$$

and $\ell|i$ and $\ell|\{i,j\}$ refer respectively to the loops in $\mathcal{L}_\mathcal{M}$ in which g^i enters and in which both g^i and g^j enter. The derivation then follows the non degenerated case, by decomposing the FIM (S.72) and factorizing out a diagonal matrix (see section SM-6.1).

Finally the complexity of the generic degenerate model reads:

$$e^{c_\mathcal{M}} = \pi^m \int_{[-1,1]^m} \mathrm{d}\gamma\, q(\gamma) \left[\prod_i \sqrt{\left(\alpha_i - \frac{\chi_i}{1+\chi}\frac{1+\gamma_i^2}{\gamma_i^2}\right)}\right] \sqrt{\det(\mathbb{1}+\mathbb{B})} \tag{S.74}$$

where the matrix $\mathbb{B}$ is defined as

$$B_{ij} = \frac{\gamma_i}{\gamma_j}\frac{1-\gamma_j^2}{1+\chi}\frac{\chi_{i,j}(1+\chi)-\chi_i\chi_j}{(\alpha_i\gamma_i^2(1+\chi)-(1+\gamma_i^2)\chi_i)} \tag{S.75}$$

and

$$q(\gamma) = \frac{1}{\pi^m}\prod_i \left(1-\gamma_i^2\right)^{-1/2}. \tag{S.76}$$

Now (S.74) is prone for standard Monte Carlo integration by random sampling γ according to the pdf $q(\gamma)$.

SM-7.3. Models with A Single Loop

We now focus on the complexity of models that contain only one loop involving all $|\mathcal{M}|$ operators, $\mathcal{L} = \{\{\emptyset\},\ell\}$, where $\ell = \{\phi^1,\ldots,\phi^{|\mathcal{M}|}\}$, parametrized by $m \leq |\mathcal{M}|$ parameters. All such models with fixed number of operators, parameters and degeneracy coefficient belong to the same class of complexity. Specifically the class of complexity is identified by the number of operators $|\mathcal{M}|$, the number of parameters m and the set of degeneracy coefficients and their multiplicities $\{(\alpha_{j_1},r_{j_1}),\ldots,(\alpha_{j_K},r_{j_K})\}$, with $\alpha_{j_1} < \ldots < \alpha_{j_K}$ (K is the total number of distinct values that the degeneracy coefficients α_i take), such that $\sum_{i=1}^K \alpha_{j_i} r_{j_i} = |\mathcal{M}|$ and $\sum_{i=1}^K r_{j_i} = m$. The cardinality of such a class of complexity is

$$\mathcal{N}_{1\,\text{loop}}^{deg} = \mathcal{N}_{1\,\text{loop}} \frac{|\mathcal{M}|!}{(\alpha_{j_1}!)^{r_{j_1}}\cdot\ldots\cdot(\alpha_{j_K}!)^{r_{j_K}}} \frac{1}{r_{j_1}!\cdot\ldots\cdot r_{j_K}!} \tag{S.77}$$

given by the number of possible ways of choosing $|\mathcal{M}|$ operators constituting a single loop $\mathcal{N}_{1\,\text{loop}}$ (S.62), times the number of partitions of a set of $|\mathcal{M}|$ elements in exactly m subsets of which r_{j_1} of cardinality α_{j_1}, ..., and r_{j_K} of cardinality α_{j_K}.

The complexity of this class of models can be derived from (S.74) by enforcing the single loop constraints on (S.73), namely $\alpha_i = \beta_i$ and $\chi_i = \alpha_i\chi$, $\chi_{i,j} = \alpha_i\alpha_j\chi$, while $\chi = \prod_{i=1}^{m} \gamma_i^{\alpha_i}$:

$$e^{c_{\mathcal{M}}} = \pi^m \int_{[-1,1]^m} \mathrm{d}\gamma\, q(\gamma) \left[\prod_i \sqrt{\left(\alpha_i - \frac{\chi}{1+\chi}\frac{1+\gamma_i^2}{\gamma_i^2}\right)}\right] \sqrt{1 + \frac{\chi}{1+\chi}\sum_i \alpha_i \frac{1-\gamma_i^2}{(\gamma^2-\chi)}} \tag{S.78}$$

where $q(\gamma)$ is defined in (S.76).

The expression (S.78) for the complexity was obtained by replacing the relation (that only holds for single loop models)

$$\det(\mathbb{1} + \mathbb{B}) = 1 + \frac{\chi}{1+\chi}\sum_i \alpha_i \frac{1-\gamma_i^2}{(\gamma^2-\chi)}. \tag{S.79}$$

in the complexity of the generic degenerate model (S.74).

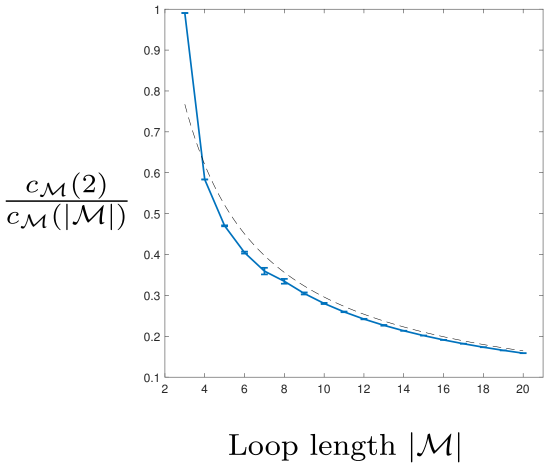

Figure S2. Degeneracy and complexity. Ratio (data points + solid line to guide the eye) between the complexity of two single loop models of length $|\mathcal{M}|$ (number of operators) parametrised respectively by 2 parameters (one of them $|\mathcal{M}| - 1$ times degenerated) and $|\mathcal{M}|$ parameters, versus loop length $|\mathcal{M}|$. Complexities here are averages of 10^3 numerical estimates of the integrals (S.66) and (S.78) using 10^6 MC samples each and error bars result from error propagation of one standard deviation of these estimates. The larger the loop the more independent are the operators, as shown in Figure 3 of the main text, such that the ratio between the complexities of single loop degenerated and non degenerated models is approaching the ratio (dashed line) between the complexities of independent operators degenerated ($\log\sqrt{|\mathcal{M}|-1}\pi^2$) and non-degenerated ($\log\pi^{|\mathcal{M}|}$) models (see SM-4.1 and SM-7.1).

The simple case of single loop models constitutes a suitable platform to gain some insights on how the degeneracy affects the complexity of models with loops. The degeneracy coefficients of parameters enter the expression of the complexity (S.78) in a non trivial way, such that numerical exploration is required. In Figure S2 we compare the complexity of a single loop model of length $|\mathcal{M}|$ (number of operators) parametrised by 2 parameters and the corresponding non degenerated single loop model. By increasing the length of the loop the degeneracy increases while the complexity decreases (relatively to the non degenerated model). The fact that—analogously to the independent operators model (see SM-7.1)—degeneracy in the single loop model reduces the complexity can be intuitively understood through our general argument in SM-4. By degenerating the parameters g^μ one indirectly constrains

the averages of the operators ϕ^μ (dual coordinates φ^μ in the model manifold) resulting in a downsized marginal polytope $\mathcal{F}$ and a smaller complexity as a consequence.

References and Notes

1. The spin s_i belongs to μ if the ith digit (starting from the right) in the binary representation of μ is 1. For example, $\phi^5(s) = s_1 s_3$, since the binary representation of 5 is $0\ldots0101$, with 1 only in the 1st and 3rd positions along the binary string of length n.
2. Mastromatteo, I. On the typical properties of inverse problems in statistical mechanics. *ArXiv* **2013**, arXiv:cond-mat.stat-mech/1311.0190.
3. Note that the set of functions $F : \mathcal{S} \to \mathbb{R}$ is a vector space provided with the sum $(F+G)(\boldsymbol{s}) = F(\boldsymbol{s}) + G(\boldsymbol{s})$ and the multiplication by scalar in $\mathbb{R}$. It is also provided with the scalar product $< F, G >= \frac{1}{2^n}\sum_{\boldsymbol{s}} F(\boldsymbol{s})G(\boldsymbol{s})$ and Ω_n as an orthonormal basis.
4. Note: with the definition of *loops*, that will be introduced in SM-2, a set of *independent operators* is a set of operators that doesn't form any loop (but the *empty loop*).
5. We will use the same notation $\mathcal{M}$ for the model, the subset of operators, or the subset of values of μ that identify the operators in the model.
6. However, let us remark that this symmetry is not preserved under the *gauge transformations* that will be introduced later, because two operators of the same order can be mapped to operators of different orders (see SM-1).
7. Rissanen, J.J. Fisher information and stochastic complexity. *IEEE trans. inf. theory* **1996**, *42*, 40–47.
8. Myung, I.J.; Balasubramanian, V.; Pitt, M.A. Counting probability distributions: Differential geometry and model selection. *Proc. Natl. Acad. Sci.* **2000**, *97*, 11170–11175.
9. Grünwald, P. *The Minimum Description Length Principle (Adaptive computation and machine learning)*; MIT Press: Cambridge, UK, 2007.
10. Jeffreys, H. An Invariant Form for the Prior Probability in Estimation Problems. *Proc. R. Soc. Lond. A Math. Phys. Eng. Sci.* **1946**, *186*, 453–461, doi:10.1098/rspa.1946.0056.
11. Rissanen, J. Stochastic complexity in learning. *J. Comp. Syst. Sci.* **1997**, *55*, 89–95.
12. Note that the set of configuration $\boldsymbol{s} = (s_1, \ldots, s_n)$ is itself a set of operators in Ω_n. So strictly speaking GTs can be defined as bijections from Ω_n to itself defined by the operation $\mu \to \mu' = \oplus_{i\in\mu}\nu_i$ for any particular choice of ν_i that realises a generating set of n.
13. We recall that a generating set of a Ω_n cannot include the identity operator (see SM-0). For this reason, it is directly excluded from the counting.
14. Landau, L.; Lifshitz, E. *Statistical Physics*, 3rd ed.; Elsevier Science: London, UK, 2013.
15. Kramers, H.A.; Wannier, G.H. Statistics of the Two-Dimensional Ferromagnet. Part II. *Physical Review* **1941**, *60*, 263–276, doi:10.1103/PhysRev.60.263.
16. Pelizzola, A. Cluster variation method in statistical physics and probabilistic graphical models. *J. Phys. A Math. Gen.* **2005**, *38*, R309–R339.
17. Excluding the identity operator that doesn't correspond to any interaction, and thus doesn't belong to any model.
18. Indeed, $\mathcal{L}$ is a subset of the set of all the partitions formed with the $|\mathcal{M}|$ operators of $\mathcal{M}$ of $\mathcal{M}$, which has a finite cardinality (given by the Bell number $B_{|\mathcal{M}|}$).
19. For example, consider the model $\mathcal{M} = \{s_1, s_2, s_3, s_1s_2, s_1s_3\}$: $\ell_1 = \{s_1, s_2, s_1s_2\}$ and $\ell_2 = \{s_1, s_3, s_1s_3\}$ are two loops of $\mathcal{M}$; $\ell_3 = \ell_1 \oplus \ell_2 = \{s_2, s_3, s_1s_2, s_1s_3\}$ is also a loop of $\mathcal{M}$. Note also that $\ell_2 = \ell_1 \oplus \ell_3$ and $\ell_1 = \ell_2 \oplus \ell_3$.
20. Obtained with the same argument than the number of gauge transformations in SM-1, see Equation (S.25).
21. Wainwright, M.J.; Jordan, M.I. Variational inference in graphical models: The view from the marginal polytope. In Proceedings of the Forty-First Annual Allerton Conference on Communication, Control, and Computing, Monticello, NY, USA, 1–3 October 2003; Volume 41, pp. 961–971.
22. Amari, S.; Nagaoka, H. *Methods of Information Geometry (Translations of mathematical monographs)*; American Mathematical Society: Providence, RI, USA, 2007.

23. Notice that here we keep indicating the number of operators with $|\mathcal{M}|$, even if for degenerate models it doesn't refer to the cardinality of a set.
24. For a loop $\ell = \{\phi^{\mu_1}, ..., \phi^{\mu_{|\ell|}}\}$ one can construct the matrix $\mathbb{U}^{\ell} = \{\mathbf{U}_{:\mu_1}, ..., \mathbf{U}_{:\mu_{|\ell|}}\}$, out of the columns of $\mathbb{U}$ in (S.10), that maps the operators in ℓ to their parameters. It follows that $\beta_i(\ell) = \sum_{j=1}^{|\ell|} U^{\ell}_{i\mu_j}$ are the degeneracy coefficients of the parameters in the submodel ℓ.
